# Menstrual disturbance associated with COVID-19 vaccines: A comprehensive systematic review and meta-analysis

**DOI:** 10.1371/journal.pone.0320162

**Published:** 2025-05-16

**Authors:** Kunchok Dorjee, R. C. Sadoff, Farima Rahimi Mansour, Sangyal Dorjee, Eli M. Binder, Maria Stetson, Regina Yuen, Hyunju Kim

**Affiliations:** 1 Center for Tuberculosis and AIDS Research, Division of Infectious Diseases, Johns Hopkins University School of Medicine, Baltimore, Maryland, United States of America; 2 Department of Epidemiology, Johns Hopkins Bloomberg School of Public Health, Baltimore, Maryland, United States of America; 3 Preventative Gynecology Research Center, Shahid Beheshti University of Medical Sciences, Tehran, Iran; 4 Adams County Health Department, Brighton, Colorado, United States of America; 5 Center for Humanitarian Health, Department of Epidemiology, Johns Hopkins Bloomberg School of Public Health, Baltimore, Maryland, United States of America; UCSI University, MALAYSIA

## Abstract

**Background:**

The relationship between COVID-19 vaccines and menstrual disturbance is unclear, in part because researchers have measured different outcomes (e.g., delays vs. changes to cycle length) with various study designs. Menstrual disruption could be a decisive factor in people’s willingness to accept the COVID-19 vaccine.

**Methods:**

We searched Medline, Embase, and Web of Science for studies investigating menstrual cycle length, flow volume, post-menopausal bleeding, and unexpected or intermenstrual bleeding. Data were analyzed using fixed-effects meta-analysis with Shore’s adjusted confidence intervals for heterogeneity.

**Findings:**

Seventeen studies with >1·9 million participants were analyzed. We found a 19% greater risk of increase in menstrual cycle length as compared to unvaccinated people or pre-vaccination time-periods (summary relative risk (sRR): 1·19; 95% CI: 1·11–1·26; n = 23,718 participants). The increase in risk was the same for Pfizer-BioNTech (sRR: 1·15; 1·05–1·27; n = 16,595) and Moderna vaccines (sRR: 1·15; 1·05–1·25; n = 7,523), similar for AstraZeneca (sRR: 1·27; 1·02–1·59; n = 532), and higher for the Janssen (sRR: 1·69; 1·14–2·52; n = 751) vaccine. In the first cycle after vaccination, length increased by <half-day (summary mean difference (sMD): 0·34 days; 0·21–0·46 days; n = 30,320) after the first dose and by 0·62 days (sMD: 0·62: 0·41–0·82; n = 17,608) after the second dose. In the second cycle after vaccination, the risk was not elevated (sMD: –0·02; –0·16–0·12; n = 18,602). The increase in risk was between 7–9% but statistically insignificant for heavier flow; 7% for post-menopausal bleeding (first dose: 1·07; 1·01–1·12; n = 1,321,268 and second dose: 1·07; 1·03–1·11; n = 1,482,884); and 16–41% for unexpected or intermenstrual bleeding (first dose: 1·16; 0·83–1·61; n = 1,303,687 and second dose: 1·41; 0·99–2·01; n = 1,390,317).

**Interpretation:**

We observed a mild increase in the risk of menstrual disturbance associated with COVID-19 vaccines. Such risks are likely clinically unmeaningful. Vaccine recipients should be appropriately counseled.

## Introduction

Numerous studies have described the occurrence of menstrual disturbance after receiving COVID-19 vaccines [1–5]. Across the studies, the reported prevalence estimates of menstrual disturbance have ranged from a low of ≤1% [5] to a high of >70% [1], making it difficult to draw meaningful epidemiological inferences. There is ongoing debate on the association of COVID-19 vaccines with menstrual disturbance, with studies showing both increased [3,4,6,7] and no disturbance [6–9]. Systematic reviews that note population-level changes in menstrual patterns have relied on prevalence rates and conflicting exclusion criteria, which do not demonstrate causality or allow for the comparison of risks among vaccine brands, doses, timeframes, or menstrual outcomes [10–13]. Various aspects of this association need to be elucidated. Different theories on underlying biological plausibility have been advanced, adding to the conundrum [14–17]. We note that COVID-19 vaccine acceptance has ranged between 59% and 92% among females of reproductive age across nations, highlighting the acute need for research and clarity on the topic [18–20]. Given the extensive reporting by the media on the topic, a continued lack of clarity can fuel further vaccine hesitancy, not just for COVID-19 vaccines but also more broadly with serious implications for the prevention and control of infectious diseases globally including future pandemics. Many studies did investigate the causal relation between COVID-19 vaccines and menstrual disturbance [3,4,6–9,15,21–24], therefore, it is possible to draw meaningful inferences from a careful review and analysis of the studies. The main objective of this systematic review and meta-analysis is to determine the association between COVID-19 vaccines and various menstrual outcomes – cycle length, flow volume, post-menopausal bleeding, and intermenstrual bleeding – disaggregated by vaccine brand and dose (first vs. second). The resulting risk differences can inform clinical guidance to menstruating and post-menopausal people who are considering inoculation for COVID-19.

## Materials and methods

### Search strategy and selection criteria

We searched Medline, Embase, Web of Science, and references of published articles to identify studies published through November 30, 2023, that investigated the risk of menstrual disturbance associated with COVID-19 vaccines. We searched for studies published between January 1, 2021, and November 30, 2023, using the search terms: ‘covid-19 vaccine’, ‘“specific covid-19 vaccine name”’, ‘menstrual disturbance’, ‘menstrual health’ and ‘menstrual cycle’ (S1 Table). We started the search on September 4, 2023, with a biweekly search thereafter and final search on November 30, 2023. Two investigators conducted title and abstract search following which all investigators reviewed the full text. No dispute was encountered in the process. Data were abstracted into an Excel sheet. The first COVID-19 vaccine to receive approval from the Food and Drug Administration (FDA; Pfizer-BioNTech) was not made publicly available until December 11, 2020. Therefore, the study timeframe covers a majority of the existing literature. The inclusion criteria are: 1) study must have reported an estimate on the risk of change in cycle length, flow volume, post-menopausal bleeding, or unexpected or intermenstrual bleeding associated with COVID-19 vaccine and 2) provided a comparative estimate of the risk of menstrual disturbance associated with COVID-19 vaccine comparing vaccinated and unvaccinated people or pre- and post-menstrual time periods or provide data for calculation of a comparative estimate. Exclusion criteria are: 1) study has reported on COVID-19 vaccine related adverse events but not on menstrual disturbance; 2) reported on menstrual disturbance but did not specify type of menstrual disturbance; and 3) reported only prevalence of menstrual disturbance among vaccinated populations and did not provide data for comparative estimates and 4) did not make mention of which COVID-19 vaccine was used (S2 Table). We adapted the Quality Assessment Tool for Observational Cohort and Cross-Sectional Studies to assess the risk of bias and the quality of the studies (S3 Table) [25]. Our quality assessment accounted for sampling bias and data collection methods in addition to adjustment for confounders (S3 Table).

For studies that reported a relative estimate, exposure variable is categorized as A) any vaccine, B) Pfizer-BioNTech, C) Moderna, D) J&J Janssen, and E) Oxford-AstraZeneca. For studies that reported an absolute risk difference in the length of menstrual cycles, vaccination is categorized by recipients of A) a first dose of any COVID-19; B) a second dose of any COVID-19 vaccine; and C) a first or second dose of any COVID-19 vaccine. These categories are defined based on the availability of the data in the studies and how the results were presented. If a study had presented the risk for both the first and second doses, the individual estimates were separately incorporated into the respective analyses.

The primary outcome consists of a change in menstrual cycle length. Menstrual cycle length is defined as the duration between the first day of one period and the first day of the next period [26]. The secondary outcomes are the risk of increased menstrual flow volume, unexpected or intermenstrual bleeding, and post-menopausal bleeding. Increase in menstrual flow volume was usually assessed by asking about the volume of menstrual flow after vaccination relative to their usual or premenstrual flow.

For self-controlled studies that reported a relative risk estimate, participants were asked if their cycle length had increased after receiving the vaccine as compared to a pre-vaccine cycle(s), or otherwise asked to report their cycle length in days before and after vaccine, with which researchers calculated the change in length using the difference between the two time periods. For the self-controlled studies that reported a risk difference, the mean differences in cycle length in days between pre- and post-vaccine periods were calculated and compared. Most studies compared the mean of three immediate pre-vaccine cycles with the vaccine dose cycle or subsequent cycles. The “vaccine dose cycle” is the first cycle after inoculation. Based on clinical relevance and available data, we conducted meta-analyses to calculate (A) the pooled relative risk of change in cycle length for all COVID-19 vaccines as well as individual vaccines, and (B) the pooled mean difference in cycle length in the vaccine dose cycle – both after the first and second doses – and for the second menstrual cycle after receiving any dose of COVID-19 vaccine.

### Data analysis

First, we calculated the summary relative risk (sRR) estimates to assess the relationship between vaccination and increased mensural cycle length. These analyses were conducted separately for 1) all vaccines (Pfizer-BioNTech, Moderna, J&J Janssen, and AstraZeneca); 2) Pfizer-BioNTech; 3) Moderna; 4) J&J Janssen; and 5) AstraZeneca vaccine. Second, we calculated summary risk differences (sRD) to assess exposure-outcome relationships for 1) mean increase in the length of the vaccine dose cycle after the first dose of any vaccine; 2) mean increase in the length of the vaccine dose cycle after receiving a second dose of any vaccine; and 3) mean increase in the length of the second cycle after *any* dose of any vaccine. Finally, we separately calculated sRR estimates for increased menstrual flow volume, unexpected/intermenstrual bleeding, and post-menopausal bleeding after receiving first or second dose of COVID-19 vaccine. For studies that reported the mean cycle length and standard errors of individual groups but did not provide the mean difference, we derived the mean difference and 95% confidence intervals from the reported values. Summary estimates were calculated using fixed-effects models [27], and we assessed heterogeneity across studies using Cochran’s Q-test ($\chi^{2}$ p value <0.10) [28] and *I*^*2*^ statistics (*I*^*2 *^*> *30%) [29]. In the presence of heterogeneity, we adjusted the 95% confidence intervals for between-study variability using the method described by Shore et al [30]. For each analysis, we used all available data without estimating or replacing missing values. Studies with missing data for certain outcomes were included in analyses where data were present, ensuring that each estimate was based on the maximum available information. We have presented the results from random effects meta-analysis as well. The meta-analysis was performed in Microsoft^®^ Excel 2023 (Microsoft Corporation, Redmond, WA). We analyzed publication bias using funnel plots and Egger’s tests.

## Results

**Fig 1:**
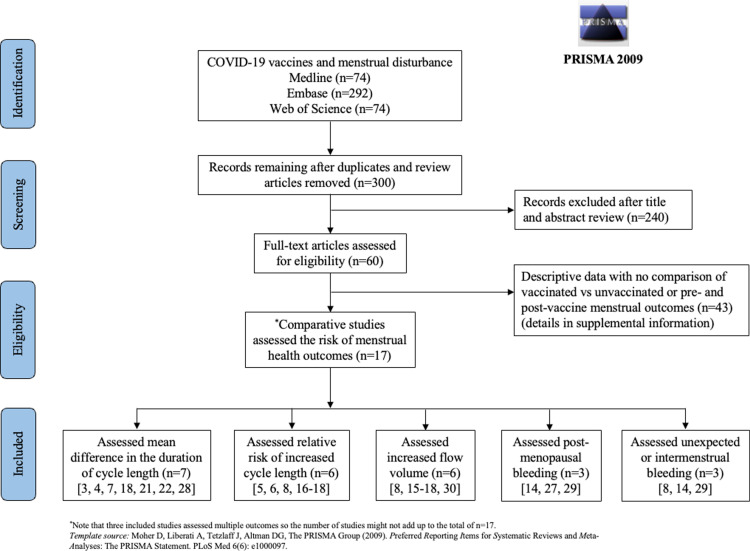
Flow diagram for selection of studies.

Our initial search yielded 440 citations. Articles were then filtered after title or abstract review, yielding 60 articles for full text review (Fig 1). Among them, 17 studies met the inclusion criteria (S2 Table). Twelve studies assessed the risk of change in cycle length, five assessed the risk of increased flow volume, three assessed the risk of post-menopausal bleeding, and three assessed the risk of unexpected or intermenstrual bleeding (S4 Table). Seven studies were from North America [3,6–8,22,24,31], seven were from Europe [15–17,21,23,32,33], one was from Asia [9], and two included participants from US, Canada, and Europe [4,34]. There were seven prospective cohort [3,4,6,7,23,24,34], four retrospective cohort [15,31–33], and five cross-sectional studies [8,9,16,17,22]. Thirteen studies used pre- and post-vaccine analysis [3,4,6,8,9,17,21–23,31–34], four studies compared vaccinated with unvaccinated populations [7,15,16,24], and four studies combined the approaches [3,4,8,34]. The studies included in the analyses are presented in Table 1, each with details about the influence of hormonal contraceptives, pre-existing gynecological conditions, prior COVID-19, and brand of COVID-19 vaccine.

**Table 1:** Key features of studies investigating the risk of menstrual disturbance associated with COVID-19 vaccines.

Author, Publication year (journal)	Place (time). Study pop. and age	Study design, sampling strategy, and sample size	Control population	Vaccines assessed and influence of vaccine brand	Menstrual outcomes reported and covariates adjusted for	Results	Influence of hormonal contraceptives	Influence of pre-existing gynecological conditions and past COVID-19
Alvergne et al., 2022 (Frontiers in Reproductive Health).	UK (Jul-Oct 2021). Menstruating individuals >18 years.	Prospective cohort and cross-sectional cohort. Internet-based sampling. Sample size: 79	Self-controlled pre- and post-vaccine analysis (SCPPVA).	AstraZeneca, Moderna. No differential effect across the two vaccine brands.	Menstrual cycle length and flow. Not adjusted for covariates.	Cycle length increased by 2.3 days after 1^st^ dose and 1.3 days after 2^nd^ dose with return to normal cycle length between the 1^st^ and 2^nd^ vaccine doses.	Delayed cycle for those using combined hormonal contraceptives. Those using only progesterone experienced heavier flow after vaccination.	Pre-existing gynecological condition is not associated with flow or timing after dose 1. After dose 2, endometriosis associated with early period.
Alvergne et al., 2023 (iScience).	UK (Mar 2021). Menstruating adults.	Cross-sectional survey. Internet-based sampling. Sample size: 11690	Unvaccinated respondents with no history of COVID-19 disease.	AstraZeneca, Pfizer-BioNTech. No differential effect across the two vaccine brands.	Cycle regularity, period duration, flow volume, intermenstrual bleeding. Contraceptives, demographics, COVID-19.	Prevalence of menstrual changes higher for women who smoke, had past COVID-19 or not using estradiol contraceptives. COVID-19 vaccine not associated with abnormal menstrual cycle overall.	Use of combined hormonal contraception was associated with lower risk of menstrual disturbance among vaccinated population.	Pre-existing gynecological conditions not associated with menstrual change after vaccine. History of COVID-19 associated with heavier, missed, and intermenstrual periods.
Blix et al., 2023 (Science advances).	Norway (Aug-Sep 2021). 32-64 years in Moba cohort and 65–80 years in the Senior Cohort.	Retrospective cohort study. Participants from 2 Norwegian population-based cohorts invited. Sample: 21,925	SCPPVA	Mainly Moderna, Pfizer-BioNTech, AstraZeneca. 30% higher risk of unexpected bleeding for Moderna than Pfizer.	Unexpected vaginal bleeding, volume, duration, episodes. Age, hormone use, BMI, educational level, gynecological condition.	The hazard ratio for postmenopausal bleeding was 3.0 after dose 1 and 2.2 after dose 2. Among those who reported it, 45% said it took place within four weeks of receiving a dose.	Incidence of menstrual disturbance was higher for those on external hormones, but hazard ratios were similar between women using and not using hormones.	No significant difference in hazard of menstrual disturbance compared between women with and without pre-existing conditions.
Bouchard et al., 2022 (Journal of Women’s Health).	USA and Canada (Not specified). Women aged 18–42.	Cross-sectional survey in two prospective cohorts. Internet-based sample (app users). Sample size: 76	SCPPVA	Pfizer-BioNTech, Moderna, J&J, AstraZeneca. Effect by vaccine brand not described.	Cycle length, menstrual volume, start date of cycle, signs of ovulation. Not adjusted for covariates.	No significant difference in cycle parameters (cycle, length, and luteal phase length, and estimated day of ovulation) between in pre-post vaccine analysis.	No difference was observed based on hormone use.	Only regularly cycling and ovulating women. Influence of past COVID-19 not described.
Caspersen et al., 2022 (Vaccine).	Norway (Mar-Aug 2020 and May-Sep 2021). Girls 12–15 years.	Prospective population-based cohort study. Cohort members invited to participate. Sample size: 7565	SCPPVA	Participants received only Pfizer-BioNTech.	Cycle length (captured as shorter or longer interval), flow volume, pain. Not adjusted for covariates.	22.6% reported any menstrual problem in the last period prior to vaccine. Heavier and prolonged bleeding higher in the cycle after vaccine. Risk of longer cycle was 15% higher (RR: 1.15 (95% CI: 1.05–1.27) after vaccine.	Participants were not using contraceptive, were not pregnant, and were not lactating.	Women with pre-existing gynecological conditions excluded. In unvaccinated girls, COVID-19 associated with greater menstrual disturbance. Association between vaccine and menstrual disturbance independent of past COVID-19.
Darney et al., 2023 (BJOG).	International (Mostly USA and Europe) (Oct 2022-May 2022). Premenopausal women 18–45 years.	Prospective cohort. Members consists of users of a menstrual tracker application. Sample size: 9555	SCPPVA as well as comparing vaccinated vs. unvaccinated.	Pfizer-BioNTech, Moderna, J&J, AstraZeneca. (Few other vaccines are included). Effect by vaccine brand not described.	Flow volume and number of days of heavy bleeding. Age, race, ethnicity, parity, BMI, education, relationship status, and global region.	Vaccinated group had higher proportion of heavier bleeding volume than the unvaccinated group. Number of heavier bleeding days did not differ between the groups.	Participants using regular hormonal contraceptives were excluded but use of emergency pills were included. Sensitivity analysis was conducted by excluding people using the emergency hormonal pills; the results were not different.	Participants with normal pre-vaccine cycle were included. Results similar in analysis excluding women with PCOS, endometriosis and thyroid disorders. Data on COVID-19 not available.
Edelman et al., 2022 (BMJ Medicine).	Global (Oct 2020-Nov 2021). Individuals aged 18–45 years.	Prospective cohort. Users of menstrual cycle tracker application. Sample size: 19622	SCPPVA as well as comparison between vaccinated and unvaccinated.	Pfizer, Moderna, AstraZeneca, Janssen, Sputnik, Covaxin, Sinopharm, & Sinovac. No difference in cycle length for vaccine brand.	Mean difference in menstrual cycle length. Age, body mass index, parity, race or ethnicity, education, relationship status, global region.	Cycle length increased by 0.71 day in the cycle that dose 1 was given and by 0.56 day in the cycle that dose 2 was given. The cycle length returned to normal in the immediate next cycle.	Participants were not using hormonal contraceptives, not pregnant, not lactating and not menopausal.	Women with pre-existing gynecological conditions excluded. Effect of past COVID-19 not described.
Edelman et al., 2022 (Obstetrics & Gynecology)	USA (Oct 2020 -Sep 2021). US residents aged 18–45 years.	Prospective cohort. Users of fertility awareness application. Sample size: 3959	SCPPVA as well as comparison between vaccinated and unvaccinated.	Pfizer, Moderna, J&J, and unspecified. Effect by vaccine brand not described.	Change in cycle length (in days). Age, race and ethnicity, BMI, education, parity, relationship status.	The difference in change in cycle length between the vaccinated and unvaccinated cohorts was < 1 day for 1^st^ or 2^nd^ dose.	Participants were not using hormonal contraceptives, not pregnant, not lactating and not menopausal.	Women with pre-existing gynecological conditions excluded. Effect of past COVID-19 not described.
Gibson et al., 2022 (NPJ Digital Health)	USA (Sep 2021-Jan 2022). Females aged ≥18 years.	Prospective cohort. Apple Women’s Cohort study. Users of menstrual tracker app. participated. Sample size: 9652	Self-controlled pre- and post-vaccine difference in cycle length	Pfizer, Moderna, Janssen, or other. Effect by vaccine brand not described.	Change in menstrual cycle length. Age, race/ethnicity, BMI, parity, and season.	Cycle length increase by <1 day. Cycle length increased by 1.26 days for J&J vaccine. Post vaccine dose cycle lengths were normal. Vaccine in follicular phase showed increased cycle length.	Participants were not using contraceptive, not pregnant, not lactating and not menopausal.	Women with pre-existing gynecological conditions excluded. Results were similar when restricted to participants without history of SARS-CoV-2 infection.
Hariton et al., 2023 (Fertility and Sterility)	USA (Mar-Jul 2022). Reproductive age women 18–55 years.	Cross-sectional. Invitation to users of a menstrual tracker app. Sample size: 5314	Self-controlled pre- and post-vaccine analysis as well as vaccinated vs. unvaccinated.	Pfizer-BioNTech, Moderna, and J&J. Effect by vaccine brand not described.	Length of cycle. Age, race, trying to conceive, symptoms.	No difference in menstrual cycle length.	Participants who used recent hormonal contraceptives were excluded.	Not specified. No difference based on SARS-CoV-2 infection.
Kajiwara et al., 2023 (Journal of Infection & Chemotherapy)	Japan (Oct 2021 – Mar 2022). Medical students aged 18–22 years.	Cross-sectional among all students of a medical institute. Sample size: 55	Comparison of menstrual outcomes: predicted and actual cycle length.	Pfizer-BioNTech or Moderna.	Regularity, cycle length. Not adjusted for covariates.	Cycle length increased by 1.6 ± 2.8 days after the 1^st^ dose and 2.5 ± 3.8 days after 2^nd^ dose.	Use of hormonal contraceptives was not specified.	Not specified. Influence of past COVID-19 not described.
Loggia et al., 2023 (Minerva Obstetrics & Gynecology)	Italy (Jan-Dec 2021). Aged 18–45 years.	Retrospective cohort. Sample size: 419	Self-controlled pre- and post-vaccine analysis.	Pfizer and Moderna. Effect by vaccine brand not described.	Cycle length, intermenstrual bleeding, and dysmenorrhea. Not adjusted for covariates.	No significant change in cycle length before and after vaccination.	Participants using hormonal contraceptives were excluded.	Not specified. Influence of past COVID-19 not described.
Ljung et al., 2023 (BMJ)	Sweden (Dec 2020-Feb 2022). Women 12–74 years old residing in Sweden.	Retrospective population-based cohort. National registry-based study. Sample: 2,946,448	Unvaccinated population compared with dose 1, 2, and dose 3.	Pfizer-BioNTech, Moderna, AstraZeneca. PMB after dose 3 higher for Pfizer followed by Moderna & AstraZeneca.	Any menstrual disturbance, pre-menopausal bleeding, and post-menopausal bleeding. Age, occupation, marital status, education, primary care/specialist visits, hospitalization, comorbidity.	Increased risk of post-menopausal bleeding (PMB), especially after the third dose for up to 90 days post vaccine. No association was observed for pre-menopausal women.	Slightly increased risk of post-menopausal bleeding after dose 3 when restricted to women without prior hormone treatment.	Not specified. Decreased menstrual disturbance including PMB in the first 7 days post-vaccine and then increased in the 8–90 days post-vaccine.
Suh-Burgmann et al., 2022 (AJOG)	USA (Dec 2020-May 2021). Females aged over 55 years.	Retrospective cohort study. Patient medical record analyzed. Sample size: 485644	Self-controlled pre- and post-vaccine analysis.	Pfizer-BioNTech, Moderna. Effect by vaccine brand not described.	Any abnormal bleeding. Not adjusted for covariates.	Post-menopausal bleeding in 0.39% of participants before vaccine, increasing to 0.47% in the first 4 months, dropping to 0.43% in the following 4 months.	Use of hormonal contraceptives was not specified.	Women with history of hysterectomy excluded. Effect of past COVID-19 not described.
Trogstad et al., 2023 (Vaccine)	Norway (May-Aug 2021). Women 18–30 years.	Population-based cohort study. Cohort members invited to participate. Sample size: 3507	Self-controlled pre- and post-vaccine analysis	Pfizer-BioNTech, Moderna. No difference based on vaccine brand observed.	Cycle length (captured as shorter or longer interval), flow volume, menstrual pain. Not adjusted for covariates.	Risk of heavier bleeding was increased after 1^st^ and 2^nd^ dose of vaccine. Risk of increased cycle length was higher post vaccine.	No significant difference in menstrual cycle disturbance was observed based on the use of hormonal contraceptives.	No significant difference in menstrual cycle disturbances based on presence of pre-existing gynecological condition. Effect of past COVID-19 not described.
Wang et al., 2022 (American Journal of Obstetrics & Gynecology)	USA and Canada (Apr 2020- Nov 2021). Nurses Health Study.	Prospective cohort. Internet-based open cohort study. Sample size: 3858	Unvaccinated.	Pfizer, Moderna, Janssen. No difference between Pfizer & Moderna. Greater cycle length increase for J&J.	Usual length of cycle and regularity. Sociodemographics, behavioral; follow-up time; pre-pandemic cycle features, infection.	Vaccinated women had higher risk of increased cycle length. The longer cycle was only in the first 6 months after vaccination.	Participants were not using hormonal contraceptives, not pregnant, not lactating and not menopausal	No difference in cycle length based on presence of pre-existing gynecological conditions. No difference in cycle length based on SARS-CoV-2 infection.
Wesselink et al., 2023 (Vaccine)	USA and Canada (Jan-Jun 2021). Females 21–45 years.	Prospective cohort study. Internet-based invitation. Sample size: 1137	Unvaccinated.	Pfizer-BioNTech, Moderna. Results were similar based on vaccine brand.	Irregular cycle, cycle length, flow volume. Age, education, health insurance type, parity, residence, follow-up.	1.1 day increase in the length of first cycle after 1st dose and 0.6 day increase in the length of the 1st cycle after 2nd dose.	Participants were not using hormonal contraceptives.	Irregular or long cycles at baseline were excluded from cycle length analysis. Results similar when restricted to women without past COVID-19.

The studies included in the meta-analysis enrolled a cumulative 1,911,755 participants (between 55 [9] and 2,946,448 [15] each). The age of the participants mostly ranged between 18 and 50. One investigation studied only adolescents 12–15 years of age [23] and two investigations included only females aged 18–30 years [9,21]. Three studies included post-menopausal women up to 80 years of age [15,31,33]. The majority of the studies included populations that were menstruating, non-pregnant, non-lactating, and not using hormonal contraceptives. Six studies reported the influence of hormonal contraceptives [15–17,21,22,33], five studies reported the influence of pre-existing gynecological conditions [7,16,17,21,33], seven studies reported the influence of past COVID-19 [6–8,15,16,23,24], and eight studies reported the influence of vaccine brand [4,7,15–17,21,24,33] (Table 1). All the studies assessed Pfizer-BioNTech vaccine, 16 studies except one [23] assessed Moderna vaccine, seven studies assessed AstraZeneca vaccine [4,15–17,22,33,34], seven studies assessed Janssen vaccine [3,4,6–8,22,34], and only two studies with very limited samples included populations that received other vaccines [4,34]. Using our quality assessment tool that assigns a study a continuous score out of 3, the average score across all 17 articles was 2.51 and none fell below 2.0 (S3 Table). The articles scored consistently well for their study designs and adjustment for confounding – averaging 2.76 and 2.59 overall, respectively and most poorly on recruitment, which averaged 1.76. The lower average score on the recruitment strategy was because many studies had employed convenience sampling to enroll the participants or surveyed a very limited population.

### Cycle length

**Fig 2:**
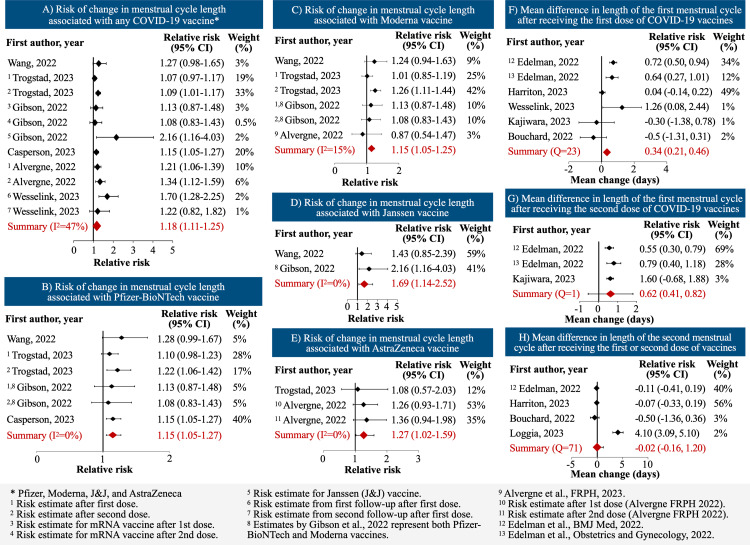
Risk of change in menstrual cycle length and mean difference in cycle length associated with specific COVID-19 vaccines and doses.

Across six studies, we observed a 19% increase in the pooled risk of increased menstrual cycle length after receiving one or two doses of COVID-19 vaccines (summary relative risk (sRR): 1.19; 95% CI: 1.11–1.26; n = 23,718). When analyzed by vaccine brand, the risk was similar for Pfizer-BioNTech (sRR: 1.15; 95% CI: 1.05–1.27; n = 16,595 participants) and Moderna (sRR: 1.15; 95% CI: 1.05–1.25; n = 7,523) vaccines. We calculated a sRR of 1.69 (95% CI: 1.14–2.52; n = 751) for J&J Janssen and 1.27 (95% CI: 1.02–1.59; n = 532) for AstraZeneca vaccine (Table 2, Fig 2). All the studies assessed the risk in the first or second menstrual cycle after vaccination except for one, which considered the risk within six months of vaccination [7]. We observed less heterogeneity when disaggregating by brand.

**Table 2:** Relative and absolute risk of menstrual disturbance associated with specific COVID-19 vaccines.

Relative risk of change in the cycle length associated with 1^st^ and 2^nd^ dose of COVID-19 vaccine										
Vaccine	No. of studies	Dose	Risk window	^#^Total sample size	Comparator population	Fixed Effects ^$^Summary relative risk; 95% CI (Shore-adjusted)			Random Effects ^$^Summary relative risk; (95% CI)	Heterogeneity *I*^*2*^; X^2^; p value
^@^Pfizer, Moderna, Johnson & Johnson, & AstraZeneca	6 [6,7,17,21,23,24]	After 1^st^ or 2^nd^ dose of vaccine	One study assessed risk in the first 6 months after vaccine. Other studies assessed risk in the first or second cycle after vaccine	23718	Unvaccinated people as well as self-controlled pre-vaccine cycles	1.19 (1.11-1.26)			1.21 (1.13-1.30)	45%; 18; p = 0.05
Pfizer	4 [6,7,21,23]	After 1^st^ or 2^nd^ dose of vaccine	One study assessed risk in the first 6 months after vaccine. Three studies assessed risk in the first or second cycle after vaccine	16595	Unvaccinated people as well as self-controlled pre-vaccine cycles	1.15 (1.05 -1.27)			N/A	0%; 2; p = 0.84
Moderna	4 [6,7,17,21]	After 1^st^ or 2^nd^ dose of vaccine	One study assessed risk in the first 6 months after vaccine. Three studies assessed risk in the first or second cycle after vaccine	7523	Unvaccinated people as well as self-controlled pre-vaccine cycles	1.15 (1.05-1.25)			N/A	15%; 6; p = 0.32
Johnson & Johnson	2 [6,7]	After 1^st^ or 2^nd^ dose of vaccine	One study assessed risk in the first 6 months after vaccine and one study assessed risk in the first cycle after vaccine.	751	Unvaccinated people and self-controlled pre-vaccine cycles	1.69 (1.14-2.52)			N/A	0%; 1; p = 0.32
AstraZeneca	2 [17,21]	After 1^st^ or 2^nd^ dose	First cycle or second cycle after vaccine	532	Self-controlled case series	1.27 (1.02-1.59)			–	0%; 0.38; p = 0.84
Mean difference in the cycle length post-vaccine (measured in days) associated with 1^st^ and 2^nd^ dose of COVID-19 vaccine										
Vaccine	No. of studies	Dose	Risk window	Total sample size	Comparator population			Pooled mean difference in cycle length (days); (95% CI)		Heterogeneity Q; p value
Pfizer-BioNTech, Moderna, J&J, & AstraZeneca	6 [3,4,8,9,22,24]	After 1^st^ dose of vaccine	The first cycle after vaccination	30320	Three studies compared vaccinated vs unvaccinated group, and three studies compared pre- and post-vaccine cycle length.			0.34 (0.21, 0.46)		0.34 (0.21, 0.46)
Pfizer-BioNTech, Moderna, J&J, & AstraZeneca	3 [3,4,9]	After 2^nd^ dose of vaccine	The first cycle after vaccination	17608	Two studies compared vaccinated vs unvaccinated group, and one study compared pre- and post-vaccine cycle length.			0.62 (0.41, 0.82)		0.62 (0.41, 0.82)
Pfizer-BioNTech and Moderna	4 [4,8,22,32]	After 1^st^ or 2^nd^ dose of vaccine	The 2^nd^ cycle after vaccination	18602	Two studies compared vaccinated vs unvaccinated group, and two studies compared pre- and post-vaccine cycle length. One study assessed the change in 3^rd^ cycle after vaccine.			-0.02 (-0.16, 0.12)		-0.02 (-0.16, 0.12)
Risk of increased flow volume, unexpected bleeding, and post-menopausal bleeding associated with COVID-19 vaccines^†^										
Outcome	No. of studies	Dose	Risk window	Total sample size	Fixed effects (shore-adjusted) Summary relative risk (95% CI)		Random Effects Summary relative risk; (95% CI)			Heterogeneity *I*^*2*^; X^2^; p value
Heavier flow volume	6 [16,17,21,23,24,34]	After 1^st^ dose of vaccine	The first or second cycle after vaccination	27544	1.07 (0.93, 1.23)		1.15 (0.91, 1.47)			96%; 160; p < 0.01
	3 [17,21,34]	After 2^nd^ dose of vaccine	The first or second cycle after vaccination	12346	1.09 (0.89, 1.34)		1.20 (0.75, 1.92)			98%; 112; p < 0.01
Post-menopausal bleeding	3 [15,31,33]	*After 1^st^ dose of vaccine	Up to 34 weeks post vaccine	1321268	1.07 (1.01, 1.12)		1.02 (0.95, 1.11)			84%; 32; p < 0.01
	3 [15,31,33]	*After 2^nd^ dose of vaccine	Up to 34 weeks post vaccine	1482884	1.07 (1.03, 1.11)		1.08 (1.02-1.14)			66%; 15; p = 0.012
Unexpected or intermenstrual bleeding	5 [15,16,21,23,33]	After 1^st^ dose of vaccine	Up to three months post vaccine	1303687	1.16 (0.83, 1.61)		1.19 (0.82, 1.72)			97%; 241; p < 0.01
	3 [15,21,33]	After 2^nd^ dose of vaccine	Up to three months post vaccine	1390317	1.41 (0.99, 2.01)		1.45 (0.95, 2.19)			97%; 127; p < 0.01

@ One study assessed risk after vaccination without specifying the dose series. The estimates are used for analysis for both first and second dose of vaccine.

# Total number of participants from the studies.

$ Pooled summary effect estimate.

*One study assessed risk after vaccination without specifying the dose series. The estimates are used for analysis for both first and second dose of vaccine.

† All studies in this section (flow volume, unexpected bleeding, and post-menopausal bleeding) compared vaccinated vs. unvaccinated groups and pre- vs. post- vaccine periods, and all assessed the following vaccine brands: Pfizer-BioNTech, Moderna, Johnson & Johnson, and AstraZeneca.

Several studies provided absolute risk difference estimates for changes to cycle length. We calculated sRD to determine the mean change in the length of the first cycle (in days) after the first and second doses of vaccine, as well as the change in the length of second cycle after first or second dose of vaccine. The change in the magnitude of risk between the first and second menstrual cycle after vaccination could indicate the risk’s reversibility over time. Across six studies in 30,320 participants, we calculated an increase of less than half a day – sRD: 0.34 (95% CI: 0.21–0.46) days – in the length of the first cycle after receiving the first dose of any of the vaccines (Table 2, Fig 2). After receiving the second dose of any of the vaccines, the length of the first cycle after vaccination increased by about two-thirds of a day (sRD: 0.62; 95% CI: 0.41–0.82 days; n = 17,608 participants). The risk of change in menstrual cycle length in the second cycle after receiving the first or second doses of Pfizer-BioNTech or Moderna vaccines was not elevated, essentially indicating a reversion of the increased risk seen in the first cycle after vaccine (sRD: -0.02; 95% CI: -0.16–0.12 days; n = 18,602). This demonstrates the importance of longitudinal studies to monitor the menstrual effects of vaccination.

For the analysis of menstrual cycle length, one study included women using contraception and with pre-existing gynecological conditions [21]. We conducted a sensitivity analysis by excluding this study. The results did not differ meaningfully (sRR: 1.18; 95% CI: 1.09–1.28). We also conducted a sensitivity analysis for the risk of increase in cycle length by excluding studies [7,21] that included women with pre-existing menstrual disturbance at baseline. The results were similar (sRR: 1.19; 95% CI: 1.12–1.27). One study had included participants that received two doses of vaccine in one menstrual cycle, but also provided estimates of cycle length increase by excluding these participants [4]. We performed sensitivity analysis using the estimates that excluded these participants. We observed a decrease but a mild, statistically significant increase in cycle length after receiving the first (sRD: 0.28; 95% CI: 0.15–0.41) and second dose (sRD: 0.42; 0.23–0.62) of COVID-19 vaccines. These sensitivity analyses allow us to confidently compare studies that have unique and possibly confounding exclusion criteria.

### Flow volume

**Fig 3:**
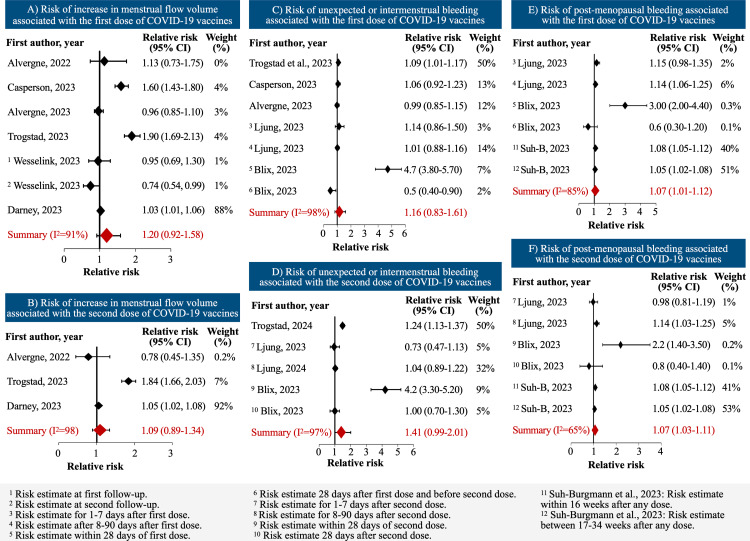
Risks of increased menstrual flow, unexpected/intermenstrual bleeding, and post-menopausal bleeding associated with COVID-19 vaccine doses.

There was a minimal and statistically insignificant increase in the risk of heavier menstrual flow volume in the first or second cycles after the first dose (sRR: 1.07; 95% CI: 0.93–1.23; n = 27,544 participants) and second dose (sRR: 1.09; 95% CI: 0.89, 1.34; n = 12,346) as compared to the pre-vaccine period or unvaccinated population (Table 2, Fig 3). Across five studies in >1.3 million people, we calculated an increase in the risk of post-menopausal bleeding associated with the first (sRR: 1.07; 95% CI: 1.01–1.12) or second dose (sRR: 1.07; 95% CI: 1.03–1.11) of COVID-19 vaccines. Risk windows are also indicated in the footnotes of the forest plot and in S4 Table.

### Unexpected/intermenstrual bleeding

Because of the limited number of studies as well as similarity of the outcomes, we combined the outcomes of unexpected bleeding and intermenstrual bleeding (Fig 3). We observed a similarly small increase in the risk after the first dose (sRR: 1.16; 95% CI: 0.83–1.61; n = 1,303,687) and a slightly greater increase in risk after the second dose of vaccine (sRR: 1.41; 95% CI: 0.99–2.01; n = 1,390,317). The risk was assessed in the first three months after vaccination. We did not find publication bias (S1 Fig), but it should be noted that some of them are self-controlled studies, which might inadvertently reveal the research question to participants.

## Discussion

The objective of this review was to characterize the relationships between various COVID-19 vaccines and measures of menstrual disturbance, including the relative risks and possible biological mechanisms of events like post-menopausal bleeding. Our analyses across 17 studies including >1.9 million participants globally revealed a minimal increase in the risk of menstrual disturbance associated with COVID-19 vaccines. We observed that there was a 19% greater risk of increase in cycle length after receiving COVID-19 vaccine. This increase was less than half a day after the first dose and two-thirds of a day after the second dose, observed only in the first cycle after vaccination, returning to normal in the second cycle. The risk was the same for Pfizer-BioNTech and Moderna vaccines (15%). Apart from cycle length, COVID-19 vaccines were associated with a 7% increase in the risk of post-menopausal bleeding, and a 7–9% increase in the risk of heavier bleeding. The results of this study are applicable for Pfizer-BioNTech, Moderna, AstraZeneca, and Janssen vaccines.

The risk estimates of the studies included in this investigation are sufficiently homogeneous. For example, of the ten relative risk estimates used to assess the cycle length increase, nine were between 1.07 and 2.16. In our analysis of the duration of cycle length increases and the reversibility of the risk, the two studies conducted by Edelman et al. carried significant weights of 34% and 12%, respectively [3,4]. These studies were well-designed, prospective in nature, and reported results from both self-controlled case series analysis and comparisons between vaccinated and unvaccinated populations. Both found an elevated risk in the first cycle after vaccination and no elevation in risk in the second cycle after vaccination. Similarly, for post-menopausal bleeding, the studies by Ljung et al. [15] and Suh-Burgmann et al. [31], which are weighed heavier in the meta-analyses, were population-based studies with sufficient power. This homogeneity distinguishes our findings from those of systematic reviews and meta-analyses that considered pooled prevalence rates rather than risk estimates [11,13].

### Biological mechanisms

The physiology underlying the menstrual disturbance associated with COVID-19 vaccines is unclear. Generally, menstruation can be affected by natural factors such as viral infections, acute stress and lifestyle factors [1,35,36]. These factors can evoke acute immunological response that can affect the menstruation. For example, we came across studies that found increased risk of menstrual disturbance following natural infection with SARS-CoV-2 [15,37,38]. Similar to these natural phenomena, vaccines may induce immune responses that affect menstruation. Of the plausible mechanisms suggested, one constitutes a vaccine-induced immune response that could transiently interfere with the hypothalamic-pituitary-ovarian (HPO) axis that controls menstruation [6,39]. COVID-19 vaccines work by producing Spike (S) proteins that bind to cellular ACE2 receptors present in various tissues including the hypothalamus, pituitary, thyroid, adrenals, ovaries, uterus, and vagina. The recognition of S proteins, particularly by CD4 + T cells, leads to the production of interferon-g and consequently, proinflammatory cytokines (PICs) (interleukin-1, IL-2, IL-6) and tumor necrosis factor (TNF)-α [40–42]. This inflammatory immune response may alter the physiological secretory function of the hypothalamus and pituitary gland to stimulate the release of corticotropin-releasing hormone (CRH) and adrenocorticotropic hormone (ACTH), respectively, the latter triggering glucocorticoid release from the adrenal glands [43]. CRH and glucocorticoids inhibit the release of gonadotropin-releasing hormone by the hypothalamus, which then slows the secretion of gonadotropins – follicle stimulating hormone and luteinizing hormone – by the anterior pituitary gland, which regulates menstruation via the ovarian sex hormones (e.g., estrogen and progesterone) [44,45]. Unfortunately, this pathway involving the PICs and HPO axis alteration may be difficult and complex for researchers to elucidate, compounded by the susceptibility of the hypothalamic mediation to disruption by lifestyle factors such as stress or sleep [43,46]. Past studies have described analogous alterations of the hypothalamus-pituitary-adrenal axis caused by cytokines [43,46].

On the other hand, a localized inflammatory immune response independent of the HPO axis is possible, as inferred by a few studies that observed (1) no difference in menstrual disturbance between women receiving and not receiving hormone replace therapy and (2) breakthrough bleeding women in menopause, whose gonads were presumably insensitive to hypothalamic-pituitary regulation [14,33]. In such a localized response, the activation of immune cells in the endometrium may affect tissue repair, leading to early shedding of the uterine lining that could explain the post-menopausal or intermenstrual bleeding [14,39]. Recently, a greater risk of menstrual cycle disturbance was observed when the COVID-19 vaccine was administered in the follicular vs. luteal phase of menstruation, supporting the theory of a mechanism that involves the disruption of the HPO axis [6].

The pathophysiology of menstrual disturbance following COVID-19 vaccination may be similar to the acute immune response observed after natural SARS-CoV-2 infection [15,37,38], although the impact of the latter may be greater and last longer [47]. Consistently higher levels of PICs in people with adverse events from COVID-19 or second doses suggest that such an immune response from COVID-19 or its vaccines may depend upon the severity of infection or the number of doses [48], which could underlie the conflicting results of positive [16,23,37,38] and negative associations [7,8,33]. This plus our observation that cycle length increase after the second dose is twice that after the first dose calls for investigation into cumulative effect. Although not emblematic of the classic dose-response phenomenon given the risk reversion after the first cycle instead of an incremental increase, a cumulative effect and a possible dose-response phenomenon is supported by evidence from a well-conducted study that showed a higher risk of menstrual disturbance when two doses were administered in a single cycle vs. one [4,7]. Our observation of decreased cycle length after excluding such participants from analysis is corroborative evidence. Similarly, greater risk of post-menopausal bleeding has been observed after the second or third dose [15]. Menstrual disturbances have also been reported after receiving typhoid [49], hepatitis B [50], and human papilloma virus vaccines [51]. As such, the existence of a shared central, localized, or combined immune response leading to the menstrual disturbance is possible.

## Strengths and limitations

To ensure validity, we pooled estimates that are clinically reasonable and undertook separate analyses for the different menstrual cycle outcomes, first and second doses, and different vaccine brands. This allowed us to generate a variety of specific weighted risk estimates for many forms of menstrual disturbance over multiple months post-vaccination. Still, because of the unavailability of data, we could not assess the menstrual outcomes other than cycle length based on vaccine brand. Because of insufficiency of data, we were not able to stratify the analysis to determine the roles of past COVID-19 or hormonal contraception for outcomes other than change in cycle length. However, as many studies included in this analysis observed the outcomes after accounting for prior COVID-19 [6–8,15,16,23,24], it is unlikely that past exposure to SARS-CoV-2 could have confounded the observed associations. Only one study investigated the relationship in girls aged 12–15 years; thus, more studies are needed to extend generalizability to this age-group.

## Future research and conclusions

Future studies must strive to determine the potential roles of: 1) underlying immune-related mechanisms; 2) exogenous hormones and differential effects based on the timing of inoculation in the follicular vs. luteal phase; 3) SARS-CoV-2 infection, stratified by disease severity as well as vaccine status; 4) vaccine components and adjuvants through translational research; and 5) long-term menstrual outcomes and reproductive health to improve vaccine technology. The studies conducted to date have shown no negative impact on fertility or reproductive health as a result of COVID-19 vaccines [52–54]. Vaccine hesitancy can rapidly reverse gains in the control of infectious diseases over the past century. In this age of social media and viral spread of information, high quality data and evidence-based policies are quintessential to allay the concerns of the public. Results of this study show that there exists but a minimal and short-lasting risk of increased menstrual disturbance associated with COVID-19 vaccines that could likely be experienced by females as a normal variant sometime during a 12-month time-frame regardless of vaccination. The fear of menstrual disturbance should not discourage anyone from getting COVID-19 vaccine.

## Supporting information

S1 Fig Assessment of publication bias. (TIF)

S1 Table Search strategy. (PDF)

S2 Table Literature search results and reasons for exclusion. (PDF)

S3 Table Quality assessment results and criteria. (PDF)

S4 Table Primary source data extraction. (PDF)

S5 Table PRISMA 2020 Checklist. (PDF)
