# Supplementary figures and images for: Menstrual disturbance associated with COVID-19 vaccines: A comprehensive systematic review and meta-analysis
Source: PLoS One. 2025 May 16;20(5):e0320162. doi: 10.1371/journal.pone.0320162 (PMC12083795; doi:10.1371/journal.pone.0320162)

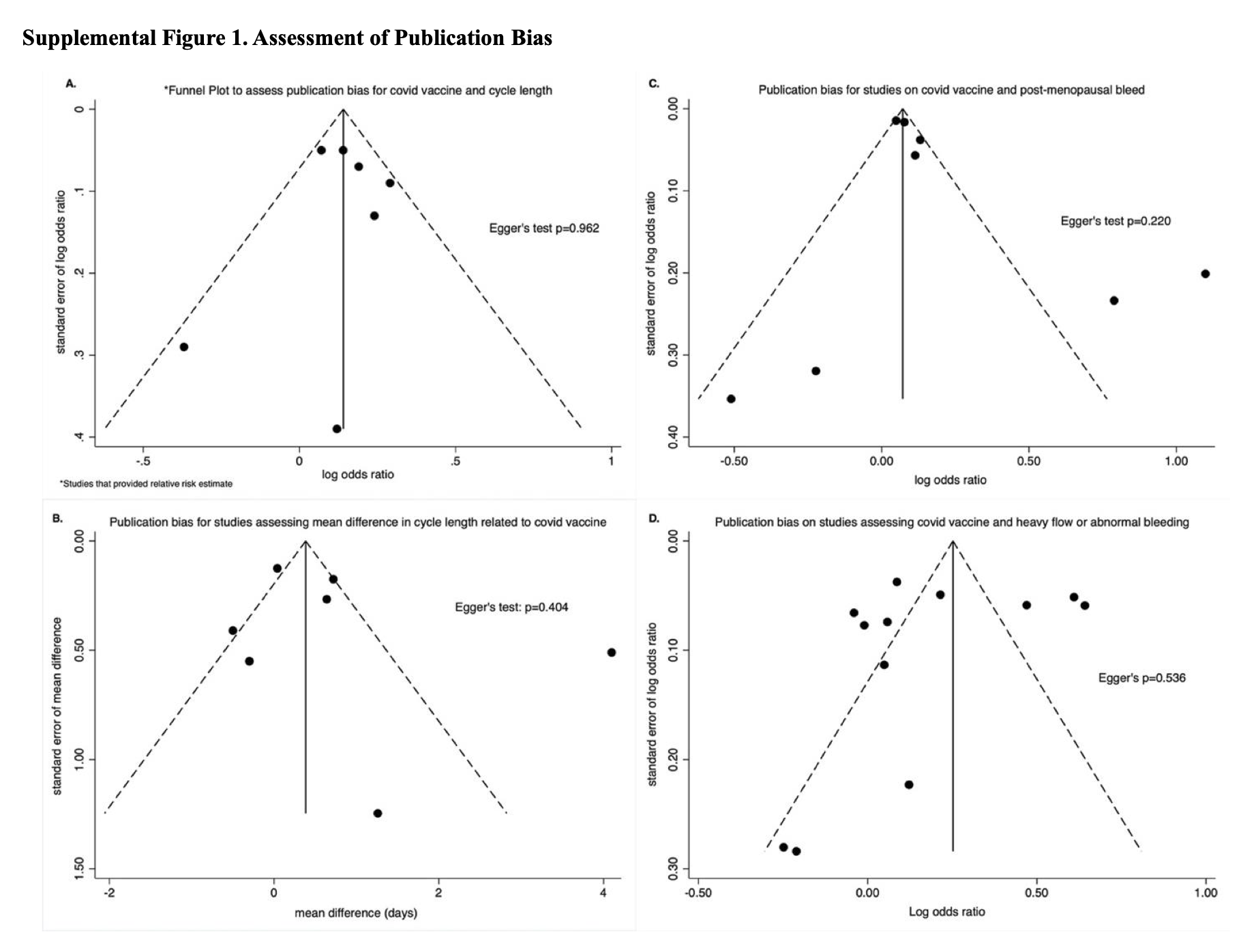

Supplement: S1 Fig — (TIF) [file pone.0320162.s001.tif]
