# Supplementary material for: Menstrual disturbance associated with COVID-19 vaccines: A comprehensive systematic review and meta-analysis
Source: PLoS One. 2025 May 16;20(5):e0320162. doi: 10.1371/journal.pone.0320162 (PMC12083795; doi:10.1371/journal.pone.0320162)
Supplement: S1 Table — (PDF) [file pone.0320162.s002.pdf]

Supplemental Table 1. Search Strategy

Search strategy for a systematic review and meta-analysis of COVID-19 vaccines and menstrual disturbance.

	Published articles from January 1, 2021 to November 30, 2023[1]
Search Terms	‘covid-19 vaccine’,<br>‘“specific covid-19 vaccine name”’,<br>‘menstrual disturbance’,<br>‘menstrual health’ and ‘menstrual cycle’ and the string ‘((covid-19 vaccine’)<br>OR (specific vaccine name(s))<br>AND ((menstrual cycle)<br>OR (menstrual bleeding)<br>OR (menstrual disturbance))’

[1] Search was conducted on September 4, 2023. A biweekly search was conducted thereafter, and final search was conducted on November 30, 2023.
