# Supplementary material for: Menstrual disturbance associated with COVID-19 vaccines: A comprehensive systematic review and meta-analysis
Source: PLoS One. 2025 May 16;20(5):e0320162. doi: 10.1371/journal.pone.0320162 (PMC12083795; doi:10.1371/journal.pone.0320162)
Supplement: S2 Table — (PDF) [file pone.0320162.s003.pdf]

Supplemental Table 2. Literature Search Results and Reasons for Exclusion

Num.	First author, publication year (journal)	Reason for exclusion
1	Abbasi, 2022 (JAMA)	Excluded during title and abstract review because the subject and/or scope of the article was clearly not applicable to our research question, so we did not conduct a full-text review.
2	Abdel-Moneim, 2022 (PLOS ONE)	Excluded during title and abstract review because the subject and/or scope of the article was clearly not applicable to our research question, so we did not conduct a full-text review.
3	Abdollahi, 2022 (International Journal of Fertility and Sterility)	Reported only prevalence of menstrual disturbance among vaccinated populations and did not provide data for comparative estimates.
4	Abdulaal, 2022 (Journal of the Bahrain Medical Society)	Excluded during title and abstract review because the subject and/or scope of the article was clearly not applicable to our research question, so we did not conduct a full-text review.
5	Abudakika, 2023 (International Journal of Health Sciences)	Excluded during title and abstract review because the subject and/or scope of the article was clearly not applicable to our research question, so we did not conduct a full-text review.
6	Abukhalil, 2023 (BMC Infectious Diseases)	Excluded during title and abstract review because the subject and/or scope of the article was clearly not applicable to our research question, so we did not conduct a full-text review.
7	Afrashtehfar, 2023 (International Journal of Environmental Research and Public Health)	Excluded during title and abstract review because the subject and/or scope of the article was clearly not applicable to our research question, so we did not conduct a full-text review.
8	Akarsu, 2022 (Vaccines)	Excluded during title and abstract review because the subject and/or scope of the article was clearly not applicable to our research question, so we did not conduct a full-text review.
9	Al-Furaydi, 2023 (European Review for Medical and Pharmacological Sciences)	Reported only prevalence of menstrual disturbance among vaccinated populations and did not provide data for comparative estimates.
10	Al-Mekhalafy, 2023 (Medical Science)	Excluded during title and abstract review because the subject and/or scope of the article was clearly not applicable to our research question, so we did not conduct a full-text review.
11	Al-Mutairi, 2022 (Vaccines)	Excluded during title and abstract review because the subject and/or scope of the article was clearly not applicable to our research question, so we did not conduct a full-text review.
12	Al-Najjar, 2022 (PLOS ONE)	Excluded during title and abstract review because the subject and/or scope of the article was clearly not applicable to our research question, so we did not conduct a full-text review.
13	Alahmadi, 2022 (Cureus)	Excluded during title and abstract review because the subject and/or scope of the article was clearly not applicable to our research question, so we did not conduct a full-text review.
14	Alamri, 2023 (Cureus)	Excluded during title and abstract review because the subject and/or scope of the article was clearly not applicable to our research question, so we did not conduct a full-text review.
15	Alblowi, 2022 (Drug Safety)	Excluded during title and abstract review because the subject and/or scope of the article was clearly not applicable to our research question, so we did not conduct a full-text review.

16	Aldali, 2022 (Saudi Med J)	Excluded during title and abstract review because the subject and/or scope of the article was clearly not applicable to our research question, so we did not conduct a full-text review.
17	Alghamdi, 2021 (Frontiers in Medicine)	Reported only prevalence of menstrual disturbance among vaccinated populations and did not provide data for comparative estimates.
18	Ali, 2022 (Infection and Drug Resistance)	Excluded during title and abstract review because the subject and/or scope of the article was clearly not applicable to our research question, so we did not conduct a full-text review.
19	Ali, 2023 (J Med Public Health)	Excluded during title and abstract review because the subject and/or scope of the article was clearly not applicable to our research question, so we did not conduct a full-text review.
20	Almomani, 2023 (Lausanne)	Excluded during title and abstract review because the subject and/or scope of the article was clearly not applicable to our research question, so we did not conduct a full-text review.
21	Almousa, 2022 (Egyptian Journal of Hospital Medicine)	Excluded during title and abstract review because the subject and/or scope of the article was clearly not applicable to our research question, so we did not conduct a full-text review.
22	Alrobaian, 2023 (Value in Health)	Excluded during title and abstract review because the subject and/or scope of the article was clearly not applicable to our research question, so we did not conduct a full-text review.
23	Alsaeedi, 2023 (Heliyon)	Excluded during title and abstract review because the subject and/or scope of the article was clearly not applicable to our research question, so we did not conduct a full-text review.
24	Alsalman, 2023 (Cureus)	Excluded during title and abstract review because the subject and/or scope of the article was clearly not applicable to our research question, so we did not conduct a full-text review.
25	Alshrouf, 2023 (Human Fertility)	Excluded during title and abstract review because the subject and/or scope of the article was clearly not applicable to our research question, so we did not conduct a full-text review.
**26**	**Alvergne, 2022 (Frontiers in Reproductive Health)**	**N/A - Study included in review.**
**27**	**Alvergne, 2023 (iScience)**	**N/A - Study included in review.**
28	Alvergne, 2023 (Trends Mol Med)	Excluded during title and abstract review because the subject and/or scope of the article was clearly not applicable to our research question, so we did not conduct a full-text review.
29	Alvergne, 2024 (Obstetrics and Gynecology)	Did not make mention of which COVID-19 vaccine was used.
30	Alzahrani, 2022 (World Family Med)	Excluded during title and abstract review because the subject and/or scope of the article was clearly not applicable to our research question, so we did not conduct a full-text review.
31	Amer, 2022 (Frontiers in Reproductive Health)	Reported only prevalence of menstrual disturbance among vaccinated populations and did not provide data for comparative estimates.
32	Anas, 2023 (Human Arenas)	Excluded during title and abstract review because the subject and/or scope of the article was clearly not applicable to our research question, so we did not conduct a full-text review.
33	Anjorin, 2022 (Vaccines)	Excluded during title and abstract review because the subject and/or scope of the article was clearly not applicable to our research question, so we did not conduct a full-text review.

34	Anto-Ocrah, 2023 (Obstet Gynecol)	Excluded during title and abstract review because the subject and/or scope of the article was clearly not applicable to our research question, so we did not conduct a full-text review.
35	Anwar, 2022 (Journal of Fatima Jinnah Medical University)	Excluded during title and abstract review because the subject and/or scope of the article was clearly not applicable to our research question, so we did not conduct a full-text review.
36	Anwar, 2023 (Medical Research Archives)	Excluded during title and abstract review because the subject and/or scope of the article was clearly not applicable to our research question, so we did not conduct a full-text review.
37	Aolymat, 2022 (Int J Environ Res Public Health)	Excluded during title and abstract review because the subject and/or scope of the article was clearly not applicable to our research question, so we did not conduct a full-text review.
38	Aolymat, 2023 (Women's Health)	Excluded during title and abstract review because the subject and/or scope of the article was clearly not applicable to our research question, so we did not conduct a full-text review.
39	Ata, 2023 (Human Reprod Update)	Excluded during title and abstract review because the subject and/or scope of the article was clearly not applicable to our research question, so we did not conduct a full-text review.
40	AUS Cohort, 2022 (Obstet Gynecol)	Excluded during title and abstract review because the subject and/or scope of the article was clearly not applicable to our research question, so we did not conduct a full-text review.
41	Azize, 2021 (Open Journal of Obstetrics and Gynecology)	Excluded during title and abstract review because the subject and/or scope of the article was clearly not applicable to our research question, so we did not conduct a full-text review.
42	Baena-García, 2022 (Women's Health)	Reported only prevalence of menstrual disturbance among vaccinated populations and did not provide data for comparative estimates.
43	Barabás, 2022 (Frontiers in Endocrinology)	Reported on menstrual disturbance but did not specify type of menstrual disturbance.
44	Beca-Martínez, 2022 (American Journal of Public Health)	Excluded during title and abstract review because the subject and/or scope of the article was clearly not applicable to our research question, so we did not conduct a full-text review.
45	Bechmann, 2022 (Horm Metab Res)	Excluded during title and abstract review because the subject and/or scope of the article was clearly not applicable to our research question, so we did not conduct a full-text review.
46	Bedi, 2023 (Indian Journal of Physiotherapy & Occupational Therapy)	Excluded during title and abstract review because the subject and/or scope of the article was clearly not applicable to our research question, so we did not conduct a full-text review.
47	Bentov, 2021 (Human Reproduction)	Reported on menstrual disturbance but did not specify type of menstrual disturbance.
48	Berkowitz, 2023 (MCN Am J Matern Child Nurs)	Excluded during title and abstract review because the subject and/or scope of the article was clearly not applicable to our research question, so we did not conduct a full-text review.
49	Berrim, 2022 (Drug Safety)	Excluded during title and abstract review because the subject and/or scope of the article was clearly not applicable to our research question, so we did not conduct a full-text review.
50	Bisgaard Jensen, 2023 (Human Reproduction)	Reported only prevalence of menstrual disturbance among vaccinated populations and did not provide data for comparative estimates.

51	Błażejewski, 2023 (J Clin Med)	Excluded during title and abstract review because the subject and/or scope of the article was clearly not applicable to our research question, so we did not conduct a full-text review.
52	Bleicher, 2021 (Vaccine)	Excluded during title and abstract review because the subject and/or scope of the article was clearly not applicable to our research question, so we did not conduct a full-text review.
**53**	**Blix, 2023 (Science Advances)**	**N/A - Study included in review.**
54	Boniface, 2023 (American Journal of Epidemiology)	Excluded during title and abstract review because the subject and/or scope of the article was clearly not applicable to our research question, so we did not conduct a full-text review.
**55**	**Bouchard, 2022 (Journal of Women's Health)**	**N/A - Study included in review.**
56	Bowman, 2021 (Reproductive Toxicology)	Reported on COVID-19 vaccine related adverse events but not on menstrual disturbance.
57	Brillo, 2022 (The Journal of Maternal-Fetal & Neonatal Medicine)	Excluded during title and abstract review because the subject and/or scope of the article was clearly not applicable to our research question, so we did not conduct a full-text review.
58	Caeran, 2022 (Drug Safety)	Excluded during title and abstract review because the subject and/or scope of the article was clearly not applicable to our research question, so we did not conduct a full-text review.
**59**	**Caspersen, 2023 (Vaccine)**	**N/A - Study included in review.**
60	Champigneulle, 2022 (Cancer Treat Res)	Excluded during title and abstract review because the subject and/or scope of the article was clearly not applicable to our research question, so we did not conduct a full-text review.
61	Chao, 2022 (Frontiers in Medicine)	Excluded during title and abstract review because the subject and/or scope of the article was clearly not applicable to our research question, so we did not conduct a full-text review.
62	Chao, 2022 (Frontiers in Reproductive Health)	Excluded during title and abstract review because the subject and/or scope of the article was clearly not applicable to our research question, so we did not conduct a full-text review.
63	Chapin-Bardales, 2021 (JAMA)	Reported on COVID-19 vaccine related adverse events but not on menstrual disturbance.
64	Chen, 2022 (Human Reproduction)	Excluded during title and abstract review because the subject and/or scope of the article was clearly not applicable to our research question, so we did not conduct a full-text review.
65	Chen, 2023 (Vaccines)	Excluded during title and abstract review because the subject and/or scope of the article was clearly not applicable to our research question, so we did not conduct a full-text review.
66	Cheng, 2022 (Human Vaccines & Immunotherapeutics)	Excluded during title and abstract review because the subject and/or scope of the article was clearly not applicable to our research question, so we did not conduct a full-text review.
67	Cheon, 2023 (PLOS ONE)	Excluded during title and abstract review because the subject and/or scope of the article was clearly not applicable to our research question, so we did not conduct a full-text review.
68	Cherenack, 2022 (PLOS ONE)	Did not make mention of which COVID-19 vaccine was used.
69	Chiang, 2023 (Physiol Rep)	Excluded during title and abstract review because the subject and/or scope of the article was clearly not applicable to our research question, so we did not conduct a full-text review.

70	Choudhary, 2021 (Lancet Infectious Diseases)	Excluded during title and abstract review because the subject and/or scope of the article was clearly not applicable to our research question, so we did not conduct a full-text review.
71	Chourasia, 2022 (Cureus)	Excluded during title and abstract review because the subject and/or scope of the article was clearly not applicable to our research question, so we did not conduct a full-text review.
72	Ciapponi, 2023 (Vaccine)	Excluded during title and abstract review because the subject and/or scope of the article was clearly not applicable to our research question, so we did not conduct a full-text review.
73	Coley, 2023 (Social Science & Medicine)	Reported on COVID-19 vaccine related adverse events but not on menstrual disturbance.
74	Dabbousi, 2023 (Irish Journal of Medical Science)	Reported only prevalence of menstrual disturbance among vaccinated populations and did not provide data for comparative estimates.
75	Dagan, 2021 (Nature Medicine)	Excluded during title and abstract review because the subject and/or scope of the article was clearly not applicable to our research question, so we did not conduct a full-text review.
76	Dar-Odeh, 2022 (Hum Vaccin Immunother)	Excluded during title and abstract review because the subject and/or scope of the article was clearly not applicable to our research question, so we did not conduct a full-text review.
**77**	**Darney, 2023 (BJOG)**	**N/A - Study included in review.**
78	Dellino, 2022 (Int J Environ Res Public Health)	Excluded during title and abstract review because the subject and/or scope of the article was clearly not applicable to our research question, so we did not conduct a full-text review.
79	Dellino, 2023 (Journal of Personalized Medicine)	Excluded during title and abstract review because the subject and/or scope of the article was clearly not applicable to our research question, so we did not conduct a full-text review.
80	Deogade, 2023 (Journal of Family Medicine and Primary Care)	Excluded during title and abstract review because the subject and/or scope of the article was clearly not applicable to our research question, so we did not conduct a full-text review.
81	DeSilva, 2023 (Obstetrics & Gynecology)	Excluded during title and abstract review because the subject and/or scope of the article was clearly not applicable to our research question, so we did not conduct a full-text review.
82	Dhalaria, 2022 (Vaccines)	Excluded during title and abstract review because the subject and/or scope of the article was clearly not applicable to our research question, so we did not conduct a full-text review.
83	Dhanani, 2022 (Fertility and Sterility)	Excluded during title and abstract review because the subject and/or scope of the article was clearly not applicable to our research question, so we did not conduct a full-text review.
84	Dorjee, 2024 (Vaccine)	Reported only prevalence of menstrual disturbance among vaccinated populations and did not provide data for comparative estimates.
85	Duijster, 2023 (British Journal of Clinical Pharmacology)	Reported only prevalence of menstrual disturbance among vaccinated populations and did not provide data for comparative estimates.
**86**	**Edelman, 2022 (BMJ Medicine)**	**N/A - Study included in review.**
**87**	**Edelman, 2022 (Obstetrics and Gynecology)**	**N/A - Study included in review.**
88	El Ayadi, 2023 (BMJ Innovations)	Excluded during title and abstract review because the subject and/or scope of the article was clearly not applicable to our research question, so we did not conduct a full-text review.

89	El-Shitany, 2022 (Int J Gen Med)	Excluded during title and abstract review because the subject and/or scope of the article was clearly not applicable to our research question, so we did not conduct a full-text review.
90	Elsebahy, 2023 (Journal of Pharmaceutical Negative Results)	Excluded during title and abstract review because the subject and/or scope of the article was clearly not applicable to our research question, so we did not conduct a full-text review.
91	Ennab, 2022 (Clinical Epidemiology and Global Health)	Excluded during title and abstract review because the subject and/or scope of the article was clearly not applicable to our research question, so we did not conduct a full-text review.
92	Fajloun, 2023 (Infectious Disorders)	Excluded during title and abstract review because the subject and/or scope of the article was clearly not applicable to our research question, so we did not conduct a full-text review.
93	Farah, 2023 (International Journal of Gynaecology and Obstetrics)	Reported only prevalence of menstrual disturbance among vaccinated populations and did not provide data for comparative estimates.
94	Farhat, 2022 (Int J Gen Med)	Excluded during title and abstract review because the subject and/or scope of the article was clearly not applicable to our research question, so we did not conduct a full-text review.
95	Farland, 2023 (Fertil Steril)	Excluded during title and abstract review because the subject and/or scope of the article was clearly not applicable to our research question, so we did not conduct a full-text review.
96	Favre, 2022 (Lancet Regional Health: Europe)	Excluded during title and abstract review because the subject and/or scope of the article was clearly not applicable to our research question, so we did not conduct a full-text review.
97	Fell, 2023 (BMJ Medicine)	Excluded during title and abstract review because the subject and/or scope of the article was clearly not applicable to our research question, so we did not conduct a full-text review.
98	Filfilan, 2023 (Cureus)	Excluded during title and abstract review because the subject and/or scope of the article was clearly not applicable to our research question, so we did not conduct a full-text review.
99	Fu, 2023 (Vaccines)	Excluded during title and abstract review because the subject and/or scope of the article was clearly not applicable to our research question, so we did not conduct a full-text review.
100	Garcia de Leon, 2023 (Women's Health)	Excluded during title and abstract review because the subject and/or scope of the article was clearly not applicable to our research question, so we did not conduct a full-text review.
101	Garg, 2022 (J South Asian Feder Obst Gynae)	Excluded during title and abstract review because the subject and/or scope of the article was clearly not applicable to our research question, so we did not conduct a full-text review.
102	Giannotta, 2023 (Vaccines)	Reported on COVID-19 vaccine related adverse events but not on menstrual disturbance.
**103**	**Gibson, 2022 (NPJ Digital Health)**	**N/A - Study included in review.**
104	Gilan, 2023 (Arch Gynecol Obstet)	Excluded during title and abstract review because the subject and/or scope of the article was clearly not applicable to our research question, so we did not conduct a full-text review.
105	Giles, 2021 (New Scientist)	Excluded during title and abstract review because the subject and/or scope of the article was clearly not applicable to our research question, so we did not conduct a full-text review.

106	Girardi, 2022 (Obstetrics & Gynecology)	Excluded during title and abstract review because the subject and/or scope of the article was clearly not applicable to our research question, so we did not conduct a full-text review.
107	Gopaul, 2023 (The Open Public Health Journal)	Excluded during title and abstract review because the subject and/or scope of the article was clearly not applicable to our research question, so we did not conduct a full-text review.
108	Gorman, 2023 (Cureus)	Excluded during title and abstract review because the subject and/or scope of the article was clearly not applicable to our research question, so we did not conduct a full-text review.
109	Goswami, 2023 (Current Medical Issues)	Excluded during title and abstract review because the subject and/or scope of the article was clearly not applicable to our research question, so we did not conduct a full-text review.
110	Guo, 2022 (Frontiers in Pharmacology)	Excluded during title and abstract review because the subject and/or scope of the article was clearly not applicable to our research question, so we did not conduct a full-text review.
111	Gupta, 2023 (Vacunas (English Edition))	Excluded during title and abstract review because the subject and/or scope of the article was clearly not applicable to our research question, so we did not conduct a full-text review.
112	Hajjo, 2023 (NPJ Vaccines)	Excluded during title and abstract review because the subject and/or scope of the article was clearly not applicable to our research question, so we did not conduct a full-text review.
113	Hallberg, 2022 (Obstetrics & Gynecology)	Excluded during title and abstract review because the subject and/or scope of the article was clearly not applicable to our research question, so we did not conduct a full-text review.
114	Hamid, 2023 (Rawal Medical Journal)	Excluded during title and abstract review because the subject and/or scope of the article was clearly not applicable to our research question, so we did not conduct a full-text review.
115	Hamza, 2022 (Vaccines)	Excluded during title and abstract review because the subject and/or scope of the article was clearly not applicable to our research question, so we did not conduct a full-text review.
116	Hantoushzadeh, 2022 (Fertil Gynecol Androl)	Excluded during title and abstract review because the subject and/or scope of the article was clearly not applicable to our research question, so we did not conduct a full-text review.
**117**	**Hariton, 2023 (Fertility and Sterility)**	**N/A - Study included in review.**
118	Harper, 2022 (International Journal of Public Health)	Excluded during title and abstract review because the subject and/or scope of the article was clearly not applicable to our research question, so we did not conduct a full-text review.
119	Harris, 2023 (JAMA)	Excluded during title and abstract review because the subject and/or scope of the article was clearly not applicable to our research question, so we did not conduct a full-text review.
120	Hasdemir, 2023 (Hum Fertil)	Excluded during title and abstract review because the subject and/or scope of the article was clearly not applicable to our research question, so we did not conduct a full-text review.
121	Hassan, 2022 (Vaccine)	Excluded during title and abstract review because the subject and/or scope of the article was clearly not applicable to our research question, so we did not conduct a full-text review.

122	Hatmal, 2022 (Vaccines)	Excluded during title and abstract review because the subject and/or scope of the article was clearly not applicable to our research question, so we did not conduct a full-text review.
123	Heidari, 2021 (Vaccines)	Excluded during title and abstract review because the subject and/or scope of the article was clearly not applicable to our research question, so we did not conduct a full-text review.
124	Helmy, 2022 (Assiut Scientific Nursing Journal)	Excluded during title and abstract review because the subject and/or scope of the article was clearly not applicable to our research question, so we did not conduct a full-text review.
125	Hillson, 2021 (Lancet)	Reported on COVID-19 vaccine related adverse events but not on menstrual disturbance.
126	Himanshi, 2022 (Asian J Pharm Clin Res)	Excluded during title and abstract review because the subject and/or scope of the article was clearly not applicable to our research question, so we did not conduct a full-text review.
127	Horowitz, 2022 (Reproductive Biomedicine Online)	Excluded during title and abstract review because the subject and/or scope of the article was clearly not applicable to our research question, so we did not conduct a full-text review.
128	Hosoya, 2022 (Obstetrics & Gynecology)	Excluded during title and abstract review because the subject and/or scope of the article was clearly not applicable to our research question, so we did not conduct a full-text review.
129	Hromić-Jahjefendić, 2023 (Vaccines)	Excluded during title and abstract review because the subject and/or scope of the article was clearly not applicable to our research question, so we did not conduct a full-text review.
130	Hu, 2022 (Frontiers in Immunology)	Excluded during title and abstract review because the subject and/or scope of the article was clearly not applicable to our research question, so we did not conduct a full-text review.
131	Hui, 2023 (American Journal of Obstetrics and Gynecology)	Excluded during title and abstract review because the subject and/or scope of the article was clearly not applicable to our research question, so we did not conduct a full-text review.
132	Issa, 2022 (Pharmacy Practice)	Excluded during title and abstract review because the subject and/or scope of the article was clearly not applicable to our research question, so we did not conduct a full-text review.
133	Issakov, 2023 (Reproductive Sciences)	Reported only prevalence of menstrual disturbance among vaccinated populations and did not provide data for comparative estimates.
134	Jacobs, 2022 (Obstetrics & Gynecology)	Excluded during title and abstract review because the subject and/or scope of the article was clearly not applicable to our research question, so we did not conduct a full-text review.
135	Jain, 2023 (Journal of Indian Academy of Clinical Medicine)	Reported only prevalence of menstrual disturbance among vaccinated populations and did not provide data for comparative estimates.
136	Jonville-Bera, 2023 (Therapies)	Excluded during title and abstract review because the subject and/or scope of the article was clearly not applicable to our research question, so we did not conduct a full-text review.
137	Joyce, 2022 (BMC Public Health)	Excluded during title and abstract review because the subject and/or scope of the article was clearly not applicable to our research question, so we did not conduct a full-text review.
138	Junkins, 2023 (Women's Health)	Excluded during title and abstract review because the subject and/or scope of the article was clearly not applicable to our research question, so we did not conduct a full-text review.

**139**	**Kajiwara, 2023 (Journal of Infection and Chemotherapy)**	**N/A - Study Included in Review.**
140	Kareem, 2022 (PLOS ONE)	Excluded during title and abstract review because the subject and/or scope of the article was clearly not applicable to our research question, so we did not conduct a full-text review.
141	Katz, 2022 (Sci Rep)	Excluded during title and abstract review because the subject and/or scope of the article was clearly not applicable to our research question, so we did not conduct a full-text review.
142	Kezia, 2022 (Int J Appl Pharm)	Excluded during title and abstract review because the subject and/or scope of the article was clearly not applicable to our research question, so we did not conduct a full-text review.
143	Khan, 2021 (American Journal of Obstetrics & Gynecology)	Excluded during title and abstract review because the subject and/or scope of the article was clearly not applicable to our research question, so we did not conduct a full-text review.
144	Khan, 2022 (American Journal of Obstetrics and Gynecology)	Reported only prevalence of menstrual disturbance among vaccinated populations and did not provide data for comparative estimates.
145	Khan, 2022 (Cumhuriyet Medical Journal)	Excluded during title and abstract review because the subject and/or scope of the article was clearly not applicable to our research question, so we did not conduct a full-text review.
146	Khan, 2023 (Women's Health)	Reported only prevalence of menstrual disturbance among vaccinated populations and did not provide data for comparative estimates.
147	Kharbanda, 2021 (JAMA Netw Open)	Excluded during title and abstract review because the subject and/or scope of the article was clearly not applicable to our research question, so we did not conduct a full-text review.
148	Kleebayoon, 2023 (Hum Fertil)	Excluded during title and abstract review because the subject and/or scope of the article was clearly not applicable to our research question, so we did not conduct a full-text review.
149	Kochhar, 2023 (Advances in Artificial Intelligence and Machine Learning)	Excluded during title and abstract review because the subject and/or scope of the article was clearly not applicable to our research question, so we did not conduct a full-text review.
150	Kolatorova, 2022 (International Journal of Molecular Sciences)	Excluded during title and abstract review because the subject and/or scope of the article was clearly not applicable to our research question, so we did not conduct a full-text review.
151	Kumar, 2024 (Current Drug Research Reviews)	Reported only prevalence of menstrual disturbance among vaccinated populations and did not provide data for comparative estimates.
152	Kumbasar, 2023 (Ginekol Pol)	Excluded during title and abstract review because the subject and/or scope of the article was clearly not applicable to our research question, so we did not conduct a full-text review.
153	Kurdoğlu, 2021 (Int J Womens Health Reprod Sci)	Excluded during title and abstract review because the subject and/or scope of the article was clearly not applicable to our research question, so we did not conduct a full-text review.
154	Laganà, 2022 (Open Medicine)	Reported only prevalence of menstrual disturbance among vaccinated populations and did not provide data for comparative estimates.
155	Laganà, 2023 (Evid Based Nurs)	Excluded during title and abstract review because the subject and/or scope of the article was clearly not applicable to our research question, so we did not conduct a full-text review.
156	Lebar, 2022 (Journal of Clinical Medicine)	Excluded during title and abstract review because the subject and/or scope of the article was clearly not applicable to our research question, so we did not conduct a full-text review.

157	Lee, 2022 (Science Advances)	Reported only prevalence of menstrual disturbance among vaccinated populations and did not provide data for comparative estimates.
158	Lee, 2023 (Clinical Nursing Research)	Title and abstract review.
159	Lessans, 2023 (International Journal of Gynaecology and Obstetrics)	Reported only prevalence of menstrual disturbance among vaccinated populations and did not provide data for comparative estimates.
160	Li, 2023 (Arch Gynecol Obstet)	Excluded during title and abstract review because the subject and/or scope of the article was clearly not applicable to our research question, so we did not conduct a full-text review.
161	Li, 2023 (J Med Virol)	Excluded during title and abstract review because the subject and/or scope of the article was clearly not applicable to our research question, so we did not conduct a full-text review.
162	Liaquat, 2022 (Ann Med Surg)	Excluded during title and abstract review because the subject and/or scope of the article was clearly not applicable to our research question, so we did not conduct a full-text review.
163	Lipkind, 2022 (MMWR)	Excluded during title and abstract review because the subject and/or scope of the article was clearly not applicable to our research question, so we did not conduct a full-text review.
164	Little, 2022 (J Clin Toxicol)	Excluded during title and abstract review because the subject and/or scope of the article was clearly not applicable to our research question, so we did not conduct a full-text review.
**165**	**Ljung, 2023 (BMJ)**	**N/A - Study included in review.**
**166**	**Loggia, 2023 (Minerva Obstetrics and Gynecology)**	**N/A - Study included in review.**
167	Lukac, 2023 (International Journal of Gynaecology and Obstetrics)	Did not make mention of which COVID-19 vaccine was used.
168	M M Al-Mehaisen, 2022 (International Journal of Women's Health)	Reported only prevalence of menstrual disturbance among vaccinated populations and did not provide data for comparative estimates.
169	Magee, 2023 (Nat Commun)	Excluded during title and abstract review because the subject and/or scope of the article was clearly not applicable to our research question, so we did not conduct a full-text review.
170	Mahasing, 2022 (Vaccines)	Excluded during title and abstract review because the subject and/or scope of the article was clearly not applicable to our research question, so we did not conduct a full-text review.
171	Maher, 2022 (Frontiers in Endocrinology)	Excluded during title and abstract review because the subject and/or scope of the article was clearly not applicable to our research question, so we did not conduct a full-text review.
172	Mahfouz, 2023 (Open Med)	Excluded during title and abstract review because the subject and/or scope of the article was clearly not applicable to our research question, so we did not conduct a full-text review.
173	Majumder, 2022 (FASEB BioAdvances)	Excluded during title and abstract review because the subject and/or scope of the article was clearly not applicable to our research question, so we did not conduct a full-text review.
174	Male, 2022 (BMJ)	Excluded during title and abstract review because the subject and/or scope of the article was clearly not applicable to our research question, so we did not conduct a full-text review.
175	Male, 2022 (Science)	Excluded during title and abstract review because the subject and/or scope of the article was clearly not applicable to our research question, so we did not conduct a full-text review.

176	Mansour, 2023 (Pharmacoepidemiology and Drug Safety)	Excluded during title and abstract review because the subject and/or scope of the article was clearly not applicable to our research question, so we did not conduct a full-text review.
177	Marcell, 2022 (American Journal of Obstetrics & Gynecology)	Excluded during title and abstract review because the subject and/or scope of the article was clearly not applicable to our research question, so we did not conduct a full-text review.
178	Mariappen, 2022 (BMJ Open)	Excluded during title and abstract review because the subject and/or scope of the article was clearly not applicable to our research question, so we did not conduct a full-text review.
179	Marques, 2022 (Women's Health)	Excluded during title and abstract review because the subject and/or scope of the article was clearly not applicable to our research question, so we did not conduct a full-text review.
180	Martignoni, 2022 (Drug Safety)	Excluded during title and abstract review because the subject and/or scope of the article was clearly not applicable to our research question, so we did not conduct a full-text review.
181	Martínez-Zamora, 2023 (Women's Health)	Excluded during title and abstract review because the subject and/or scope of the article was clearly not applicable to our research question, so we did not conduct a full-text review.
182	Matar, 2023 (Influenza Other Respir Viruses)	Excluded during title and abstract review because the subject and/or scope of the article was clearly not applicable to our research question, so we did not conduct a full-text review.
183	Medina-Perucha, 2022 (International Journal of Women's Health)	Excluded during title and abstract review because the subject and/or scope of the article was clearly not applicable to our research question, so we did not conduct a full-text review.
184	Melekhova, 2021 (Obstetrics and Gynecology)	Excluded during title and abstract review because the subject and/or scope of the article was clearly not applicable to our research question, so we did not conduct a full-text review.
185	Merchant, 2021 (BMJ)	Excluded during title and abstract review because the subject and/or scope of the article was clearly not applicable to our research question, so we did not conduct a full-text review.
186	Micallef, 2023 (Drug Saf)	Excluded during title and abstract review because the subject and/or scope of the article was clearly not applicable to our research question, so we did not conduct a full-text review.
187	Minakshi, 2022 (Front Immunol)	Excluded during title and abstract review because the subject and/or scope of the article was clearly not applicable to our research question, so we did not conduct a full-text review.
188	Mínguez-Esteban, 2022 (Biology (Basel))	Excluded during title and abstract review because the subject and/or scope of the article was clearly not applicable to our research question, so we did not conduct a full-text review.
189	Mitra, 2023 (PLOS ONE)	Excluded during title and abstract review because the subject and/or scope of the article was clearly not applicable to our research question, so we did not conduct a full-text review.
190	Moeed, 2022 (Frontiers in Public Health)	Excluded during title and abstract review because the subject and/or scope of the article was clearly not applicable to our research question, so we did not conduct a full-text review.
191	Mohammed, 2023 (Cureus)	Excluded during title and abstract review because the subject and/or scope of the article was clearly not applicable to our research question, so we did not conduct a full-text review.

192	Mohr-Sasson, 2023 (Arch Gynecol Obstet)	Excluded during title and abstract review because the subject and/or scope of the article was clearly not applicable to our research question, so we did not conduct a full-text review.
193	Moolamalla, 2022 (Journal of South Asian Federation of Obstetrics and Gynaecology)	Excluded during title and abstract review because the subject and/or scope of the article was clearly not applicable to our research question, so we did not conduct a full-text review.
194	Moro, 2022 (Vaccine)	Excluded during title and abstract review because the subject and/or scope of the article was clearly not applicable to our research question, so we did not conduct a full-text review.
195	Morris, 2021 (F&S Reports)	Reported on COVID-19 vaccine related adverse events but not on menstrual disturbance.
196	Morsi, 2022 (Egyptian Journal of Hospital Medicine)	Excluded during title and abstract review because the subject and/or scope of the article was clearly not applicable to our research question, so we did not conduct a full-text review.
197	Mose, 2022 (PLOS ONE)	Excluded during title and abstract review because the subject and/or scope of the article was clearly not applicable to our research question, so we did not conduct a full-text review.
198	Mosini, 2022 (Drug Safety)	Excluded during title and abstract review because the subject and/or scope of the article was clearly not applicable to our research question, so we did not conduct a full-text review.
199	Muhaidat, 2022 (International Journal of Women's Health)	Reported only prevalence of menstrual disturbance among vaccinated populations and did not provide data for comparative estimates.
200	Muharam, 2022 (PLOS ONE)	Excluded during title and abstract review because the subject and/or scope of the article was clearly not applicable to our research question, so we did not conduct a full-text review.
201	Nabatchian, 2023 (Lab Med)	Excluded during title and abstract review because the subject and/or scope of the article was clearly not applicable to our research question, so we did not conduct a full-text review.
202	Namiki, 2022 (The Journal of Obstetrics and Gynaecology Research)	Reported only prevalence of menstrual disturbance among vaccinated populations and did not provide data for comparative estimates.
203	Nguyen, 2022 (Preventive Medicine)	Reported on COVID-19 vaccine related adverse events but not on menstrual disturbance.
204	Noh, 2023 (Epidemiology and Health)	Excluded during title and abstract review because the subject and/or scope of the article was clearly not applicable to our research question, so we did not conduct a full-text review.
205	Nouman, 2022 (Journal of Microbiology and Molecular Genetics)	Excluded during title and abstract review because the subject and/or scope of the article was clearly not applicable to our research question, so we did not conduct a full-text review.
206	Oosterhuis, 2023 (Drug Safety)	Excluded during title and abstract review because the subject and/or scope of the article was clearly not applicable to our research question, so we did not conduct a full-text review.
207	Orient, 2022 (J Am Phys Surg)	Excluded during title and abstract review because the subject and/or scope of the article was clearly not applicable to our research question, so we did not conduct a full-text review.
208	Orvieto, 2021 (Reproductive biology and endocrinology)	Reported on COVID-19 vaccine related adverse events but not on menstrual disturbance.

209	Ozimek, 2022 (J Women's Health)	Excluded during title and abstract review because the subject and/or scope of the article was clearly not applicable to our research question, so we did not conduct a full-text review.
210	Paik, 2023 (Clin Exp Reprod Med)	Excluded during title and abstract review because the subject and/or scope of the article was clearly not applicable to our research question, so we did not conduct a full-text review.
211	Pandit, 2022 (Cureus)	Excluded during title and abstract review because the subject and/or scope of the article was clearly not applicable to our research question, so we did not conduct a full-text review.
212	Pardo-Cabello, 2023 (European Journal of Internal Medicine)	Excluded during title and abstract review because the subject and/or scope of the article was clearly not applicable to our research question, so we did not conduct a full-text review.
213	Park, 2023 (J Med Internet Res)	Excluded during title and abstract review because the subject and/or scope of the article was clearly not applicable to our research question, so we did not conduct a full-text review.
214	Patel, 2023 (Methods of Information in Medicine)	Excluded during title and abstract review because the subject and/or scope of the article was clearly not applicable to our research question, so we did not conduct a full-text review.
215	Payne, 2022 (International Journal of Environmental Research and Public Health)	Excluded during title and abstract review because the subject and/or scope of the article was clearly not applicable to our research question, so we did not conduct a full-text review.
216	Payne, 2023 (Vaccine)	Excluded during title and abstract review because the subject and/or scope of the article was clearly not applicable to our research question, so we did not conduct a full-text review.
217	Petruk, 2023 (Wiad Lek)	Excluded during title and abstract review because the subject and/or scope of the article was clearly not applicable to our research question, so we did not conduct a full-text review.
218	Pezzaioli, 2022 (Frontiers in Endocrinology)	Excluded during title and abstract review because the subject and/or scope of the article was clearly not applicable to our research question, so we did not conduct a full-text review.
219	Phelan, 2021 (Frontiers in Endocrinology)	Excluded during title and abstract review because the subject and/or scope of the article was clearly not applicable to our research question, so we did not conduct a full-text review.
220	Pietri, 2022 (Human Reproduction)	Excluded during title and abstract review because the subject and/or scope of the article was clearly not applicable to our research question, so we did not conduct a full-text review.
221	Pollack, 2023 (Front Rehabil Sci)	Excluded during title and abstract review because the subject and/or scope of the article was clearly not applicable to our research question, so we did not conduct a full-text review.
222	Pourmasumi, 2023 (Balkan Medical Journal)	Excluded during title and abstract review because the subject and/or scope of the article was clearly not applicable to our research question, so we did not conduct a full-text review.
223	Prado, 2021 (Health Science Reports)	Excluded during title and abstract review because the subject and/or scope of the article was clearly not applicable to our research question, so we did not conduct a full-text review.
224	Priya, 2022 (Biomedicine)	Excluded during title and abstract review because the subject and/or scope of the article was clearly not applicable to our research question, so we did not conduct a full-text review.

225	Qashqari, 2022 (Ethiop J Health Sci)	Excluded during title and abstract review because the subject and/or scope of the article was clearly not applicable to our research question, so we did not conduct a full-text review.
226	Qawaqzeh, 2022 (International Journal of Medical Students)	Excluded during title and abstract review because the subject and/or scope of the article was clearly not applicable to our research question, so we did not conduct a full-text review.
227	Qazi, 2023 (Int J Gynaecol Obstet)	Excluded during title and abstract review because the subject and/or scope of the article was clearly not applicable to our research question, so we did not conduct a full-text review.
228	Rahimi Mansour, 2023 (Journal of Reproductive Immunology)	Reported only prevalence of menstrual disturbance among vaccinated populations and did not provide data for comparative estimates.
229	Rastegar, 2023 (New Microbes New Infect)	Excluded during title and abstract review because the subject and/or scope of the article was clearly not applicable to our research question, so we did not conduct a full-text review.
230	Raz, 2021 (Vaccine)	Excluded during title and abstract review because the subject and/or scope of the article was clearly not applicable to our research question, so we did not conduct a full-text review.
231	Razzaghi, 2021 (MMWR)	Excluded during title and abstract review because the subject and/or scope of the article was clearly not applicable to our research question, so we did not conduct a full-text review.
232	Riccardi, 2022 (Drug Safety)	Excluded during title and abstract review because the subject and/or scope of the article was clearly not applicable to our research question, so we did not conduct a full-text review.
233	Ricke, 2022 (Med Hypotheses)	Excluded during title and abstract review because the subject and/or scope of the article was clearly not applicable to our research question, so we did not conduct a full-text review.
234	Rivlin, 2022 (Internal Medicine Alert)	Excluded during title and abstract review because the subject and/or scope of the article was clearly not applicable to our research question, so we did not conduct a full-text review.
235	Rodríguez Quejada, 2022 (Women's Health)	Reported on menstrual disturbance but did not specify type of menstrual disturbance.
236	Rodríguez Quejada, 2022 (Women's Health)	Reported on COVID-19 vaccine related adverse events but not on menstrual disturbance.
237	Rogers, 2022 (BMJ Open)	Excluded during title and abstract review because the subject and/or scope of the article was clearly not applicable to our research question, so we did not conduct a full-text review.
238	Rohatgi, 2023 (Frontiers in Global Women's Health)	Excluded during title and abstract review because the subject and/or scope of the article was clearly not applicable to our research question, so we did not conduct a full-text review.
239	Romero-Rodríguez, 2022 (Frontiers in Medicine)	Excluded during title and abstract review because the subject and/or scope of the article was clearly not applicable to our research question, so we did not conduct a full-text review.
240	Romero, 2023 (International Journal of Vaccine Theory, Practice, and Research)	Excluded during title and abstract review because the subject and/or scope of the article was clearly not applicable to our research question, so we did not conduct a full-text review.
241	Roncati, 2022 (Brain Hemorrhages)	Excluded during title and abstract review because the subject and/or scope of the article was clearly not applicable to our research question, so we did not conduct a full-text review.

242	Roncati, 2023 (Brain Hemorrhages)	Excluded during title and abstract review because the subject and/or scope of the article was clearly not applicable to our research question, so we did not conduct a full-text review.
243	Saadedine, 2023 (Biology of Reproduction)	Excluded during title and abstract review because the subject and/or scope of the article was clearly not applicable to our research question, so we did not conduct a full-text review.
244	Sabat, 2023 (Frontiers in Immunology)	Excluded during title and abstract review because the subject and/or scope of the article was clearly not applicable to our research question, so we did not conduct a full-text review.
245	Sachdeva, 2023 (International Journal of Preclinical & Clinical Research)	Excluded during title and abstract review because the subject and/or scope of the article was clearly not applicable to our research question, so we did not conduct a full-text review.
246	Saçıntı, 2022 (J Obstet Gynaecol)	Excluded during title and abstract review because the subject and/or scope of the article was clearly not applicable to our research question, so we did not conduct a full-text review.
247	Sadat Larijani, 2023 (Pathogens and Disease)	Excluded during title and abstract review because the subject and/or scope of the article was clearly not applicable to our research question, so we did not conduct a full-text review.
248	Saleem, 2022 (J Pak Med Assoc)	Excluded during title and abstract review because the subject and/or scope of the article was clearly not applicable to our research question, so we did not conduct a full-text review.
249	Saleh Alzahrani, 2023 (Saudi Pharm J)	Excluded during title and abstract review because the subject and/or scope of the article was clearly not applicable to our research question, so we did not conduct a full-text review.
250	Sarfraz, 2022 (Annals of Medicine and)	Reported only prevalence of menstrual disturbance among vaccinated populations and did not provide data for comparative estimates.
251	SC van der Boor, 2023 (Vaccine)	Excluded during title and abstract review because the subject and/or scope of the article was clearly not applicable to our research question, so we did not conduct a full-text review.
252	Seo, 2022 (Dev Reprod)	Excluded during title and abstract review because the subject and/or scope of the article was clearly not applicable to our research question, so we did not conduct a full-text review.
253	Seyfi-Ghale-Jogh, 2023 (Journal of Obstetrics, Gynecology and Cancer Research)	Excluded during title and abstract review because the subject and/or scope of the article was clearly not applicable to our research question, so we did not conduct a full-text review.
254	Sharma, 2023 (Journal of South Asian Federation of Obstetrics and Gynaecology)	Excluded during title and abstract review because the subject and/or scope of the article was clearly not applicable to our research question, so we did not conduct a full-text review.
255	Sharp, 2022 (International Journal of Epidemiology)	Excluded during title and abstract review because the subject and/or scope of the article was clearly not applicable to our research question, so we did not conduct a full-text review.
256	Shimabukuro, 2021 (NEJM)	Excluded during title and abstract review because the subject and/or scope of the article was clearly not applicable to our research question, so we did not conduct a full-text review.
257	Skelly, 2021 (Nature Communications)	Reported on COVID-19 vaccine related adverse events but not on menstrual disturbance.

258	Soltani Hekmat, 2021 (Biomed Res Int)	Excluded during title and abstract review because the subject and/or scope of the article was clearly not applicable to our research question, so we did not conduct a full-text review.
259	Soysal, 2022 (Saudi Medical Journal)	Excluded during title and abstract review because the subject and/or scope of the article was clearly not applicable to our research question, so we did not conduct a full-text review.
260	Stein, 2022 (Vaccines)	Excluded during title and abstract review because the subject and/or scope of the article was clearly not applicable to our research question, so we did not conduct a full-text review.
261	Stochino, 2022 (Drug Safety)	Excluded during title and abstract review because the subject and/or scope of the article was clearly not applicable to our research question, so we did not conduct a full-text review.
262	Sualeh, 2022 (Cureus)	Reported only prevalence of menstrual disturbance among vaccinated populations and did not provide data for comparative estimates.
**263**	**Suh-Burgmann, 2022 (American Journal of Obstetrics and Gynecology)**	**N/A - Study included in review.**
264	Sun, 2023 (BMC Women's Health)	Excluded during title and abstract review because the subject and/or scope of the article was clearly not applicable to our research question, so we did not conduct a full-text review.
265	Syed, 2023 (Cureus)	Excluded during title and abstract review because the subject and/or scope of the article was clearly not applicable to our research question, so we did not conduct a full-text review.
266	Taşkaldıran, 2022 (Int J Clin Pract)	Excluded during title and abstract review because the subject and/or scope of the article was clearly not applicable to our research question, so we did not conduct a full-text review.
267	Taylor, 2023 (BMJ)	Excluded during title and abstract review because the subject and/or scope of the article was clearly not applicable to our research question, so we did not conduct a full-text review.
268	Tayyaba Rehan, 2022 (Health Science Reports)	Excluded during title and abstract review because the subject and/or scope of the article was clearly not applicable to our research question, so we did not conduct a full-text review.
269	Thabet, 2023 (Egyptian Journal of Health Care)	Excluded during title and abstract review because the subject and/or scope of the article was clearly not applicable to our research question, so we did not conduct a full-text review.
270	Thorp, 2023 (Human Reproduction)	Excluded during title and abstract review because the subject and/or scope of the article was clearly not applicable to our research question, so we did not conduct a full-text review.
271	Trogstad, 2022 (BMJ Med)	Excluded during title and abstract review because the subject and/or scope of the article was clearly not applicable to our research question, so we did not conduct a full-text review.
**272**	**Trogstad, 2023 (Vaccine)**	**N/A - Study included in review.**
273	Tsukahara, 2022 (Sports)	Title and abstract review.
274	Tsundue, 2022 (BMJ global health)	Reported on COVID-19 vaccine related adverse events but not on menstrual disturbance.
275	Valnet-Rabier, 2023 (Therapie)	Excluded during title and abstract review because the subject and/or scope of the article was clearly not applicable to our research question, so we did not conduct a full-text review.

276	Vassallo, 2021 (Frontiers in Global Women's Health)	Excluded during title and abstract review because the subject and/or scope of the article was clearly not applicable to our research question, so we did not conduct a full-text review.
277	Velasco-Regulez, 2022 (Am J Obstet Gynecol)	Excluded during title and abstract review because the subject and/or scope of the article was clearly not applicable to our research question, so we did not conduct a full-text review.
278	Verkerk, 2022 (Journal of Vaccine Theory)	Excluded during title and abstract review because the subject and/or scope of the article was clearly not applicable to our research question, so we did not conduct a full-text review.
279	Vernon, 2023 (J Am Pharm Assoc)	Excluded during title and abstract review because the subject and/or scope of the article was clearly not applicable to our research question, so we did not conduct a full-text review.
280	Walcherberger, 2022 (European Journal of Politics and Gender)	Excluded during title and abstract review because the subject and/or scope of the article was clearly not applicable to our research question, so we did not conduct a full-text review.
281	Wali, 2023 (Cureus)	Excluded during title and abstract review because the subject and/or scope of the article was clearly not applicable to our research question, so we did not conduct a full-text review.
**282**	**Wang, 2022 (American Journal of Obstetrics and Gynecology)**	**N/A - Study included in review.**
283	Weinberg, 2023 (Am J Epidemiol)	Excluded during title and abstract review because the subject and/or scope of the article was clearly not applicable to our research question, so we did not conduct a full-text review.
284	Weinerman, 2022 (Fertility and Sterility)	Excluded during title and abstract review because the subject and/or scope of the article was clearly not applicable to our research question, so we did not conduct a full-text review.
**285**	**Wesselink, 2023 (Vaccine)**	**N/A - Study included in review.**
286	Wong, 2022 (Lancet Digital Health)	Reported only prevalence of menstrual disturbance among vaccinated populations and did not provide data for comparative estimates.
287	Xu, 2021 (Clinical and Translational Medicine)	Excluded during title and abstract review because the subject and/or scope of the article was clearly not applicable to our research question, so we did not conduct a full-text review.
288	Yacoub, 2022 (Gynecol Endocrinol)	Excluded during title and abstract review because the subject and/or scope of the article was clearly not applicable to our research question, so we did not conduct a full-text review.
289	Yang, 2023 (JAMA Network)	Excluded during title and abstract review because the subject and/or scope of the article was clearly not applicable to our research question, so we did not conduct a full-text review.
290	Yasmin, 2021 (Frontiers in Public Health)	Excluded during title and abstract review because the subject and/or scope of the article was clearly not applicable to our research question, so we did not conduct a full-text review.
291	Yazdani, 2023 (Vaccines)	Excluded during title and abstract review because the subject and/or scope of the article was clearly not applicable to our research question, so we did not conduct a full-text review.
292	Yoosefian, 2023 (Journal of Public Health)	Excluded during title and abstract review because the subject and/or scope of the article was clearly not applicable to our research question, so we did not conduct a full-text review.

293	Zaçe, 2022 (Vaccine)	Excluded during title and abstract review because the subject and/or scope of the article was clearly not applicable to our research question, so we did not conduct a full-text review.
294	Zaher, 2023 (Biomedicines)	Excluded during title and abstract review because the subject and/or scope of the article was clearly not applicable to our research question, so we did not conduct a full-text review.
295	Zauche, 2021 (NEJM)	Excluded during title and abstract review because the subject and/or scope of the article was clearly not applicable to our research question, so we did not conduct a full-text review.
296	Zechiu, 2022 (Ro J Infect Dis)	Excluded during title and abstract review because the subject and/or scope of the article was clearly not applicable to our research question, so we did not conduct a full-text review.
297	Zhang, 2022 (BMC women's health)	Reported only prevalence of menstrual disturbance among vaccinated populations and did not provide data for comparative estimates.
298	Zhao, 2023 (Journal of Medical Virology)	Excluded during title and abstract review because the subject and/or scope of the article was clearly not applicable to our research question, so we did not conduct a full-text review.
299	Zhong, 2023 (BMC Women's Health)	Excluded during title and abstract review because the subject and/or scope of the article was clearly not applicable to our research question, so we did not conduct a full-text review.
300	Zhou, 2023 (Digital Health)	Excluded during title and abstract review because the subject and/or scope of the article was clearly not applicable to our research question, so we did not conduct a full-text review.
