# Supplementary material for: Menstrual disturbance associated with COVID-19 vaccines: A comprehensive systematic review and meta-analysis
Source: PLoS One. 2025 May 16;20(5):e0320162. doi: 10.1371/journal.pone.0320162 (PMC12083795; doi:10.1371/journal.pone.0320162)
Supplement: S3 Table — (PDF) [file pone.0320162.s004.pdf]

**Supplemental Table 3. Quality Assessment Results and Criteria**

Adapted from the National Institutes of Health's Quality Assessment Tool for Observational Cohort and Cross-Sectional Studies.[1] Point system: 3=good, 2=fair, 1=poor.

| First author, year (journal) | 1) Study design | 2) Population | 3) Recruitment | 4) Justification | 5) Timeframe | 6) Outcome variable | 7) Validity, reliability | 8) Confounders | Average score |
|---|---|---|---|---|---|---|---|---|---|
| Alvergne, 2022 (Frontiers in Reproductive Health) | 3 | 3 | 3 | 3 | 3 | 3 | 3 | 3 | 3.0 |
| Alvergne, 2023 (iScience) | 3 | 2 | 3 | 3 | 3 | 3 | 3 | 3 | 2.9 |
| Blix, 2022 (Science Advances) | 3 | 3 | 3 | 3 | 3 | 3 | 2 | 3 | 2.9 |
| Bouchard, 2022 (Journal of Women's Health) | 2 | 3 | 1 | 2 | 3 | 2 | 3 | 3 | 2.4 |
| Caspersen, 2022 (Vaccine) | 3 | 3 | 1 | 3 | 3 | 2 | 1 | 1 | 2.1 |
| Darney et al., 2023 (BJOG) | 3 | 3 | 1 | 2 | 3 | 2 | 2 | 3 | 2.4 |
| Edelman, 2022 (BMJ Medicine) | 3 | 3 | 1 | 2 | 3 | 2 | 2 | 3 | 2.4 |
| Edelman, 2022 (Obstetrics & Gynecology) | 3 | 3 | 1 | 2 | 3 | 3 | 3 | 3 | 2.6 |
| Gibson, 2022 (NPJ Digital Health) | 3 | 3 | 1 | 3 | 3 | 3 | 3 | 3 | 2.8 |
| Hariton, 2023 (Fertility and Sterility) | 3 | 3 | 1 | 1 | 3 | 1 | 3 | 2 | 2.1 |
| Kajiwara, 2023 (Journal of Infection and Chemotherapy) | 2 | 3 | 1 | 3 | 3 | 2 | 2 | 2 | 2.3 |
| Ljung, 2023 (BMJ) | 2 | 3 | 3 | 2 | 3 | 3 | 3 | 3 | 2.8 |
| Loggia et al., 2023 (Minerva Obstetrics & Gynecology) | 2 | 3 | 2 | 1 | 3 | 3 | 2 | 1 | 2.1 |
| Suh-Burgmann, 2022 (American Journal of Obstetrics & Gynecology) | 3 | 3 | 1 | 1 | 3 | 2 | 3 | 2 | 2.3 |
| Trogstad, 2023 (Vaccine) | 3 | 3 | 3 | 2 | 3 | 3 | 2 | 3 | 2.8 |
| Wang, 2022 (American Journal of Obstetrics & Gynecology) | 3 | 2 | 1 | 1 | 3 | 3 | 2 | 3 | 2.3 |
| Wesselink, 2023 (Vaccine) | 3 | 3 | 3 | 3 | 3 | 3 | 2 | 3 | 2.9 |

Quality Assessment Criteria

1. **Was the study design appropriate to this research question?**
    (1) No (e.g., only descriptive, unknown)
    (2) Somewhat (e.g., cross-sectional survey (one group, one time), interrupted time series)
    (3) Yes (e.g., randomized control trial, prospective and/or cohort analytic (two groups, pre-post))

[1] National Heart, Lung, and Blood Institute. "Study Quality Assessment Tools." *National Institutes of Health*, 2013. Available from: https://www.nhlbi.nih.gov/health-topics/study-quality-assessment-tools

2. **Was the study population clearly specified and defined?**
   (1) No
   (2) Yes, broadly (e.g., "women of reproductive age")
   (3) Yes, with definition (e.g., "females aged 18-45 years")

3. **Were all the subjects selected or recruited from the same or similar populations (including the same time period)?**
   (1) Only used an exclusive population (e.g., users of a specific app, healthcare workers in a specific hospital)
   (2) Used a convenience sampling strategy (e.g., peer referrals)
   (3) Used a representative/robust sampling strategy (e.g., online advertisements)

4. **Was a sample size justification, power description, or variance and *effect estimates* provided?**
   (1) No, there was only prevalence/incidence
   (2) Yes, there is prevalence/incidence and one or more of the following: sample size justification, power description, or variance
   (3) Yes, there is an effect estimate and something else (e.g., prevalence)

5. **Was the timeframe sufficient so that one could reasonably expect to see an association between exposure and outcome if it existed?**
   (1) No, only one measurement was taken within seven days
   (2) At least one measurement was taken after seven days
   (3) Multiple measurements were taken (including at least one after seven days)

6. **Did the study examine different levels of the outcome (e.g., categories of exposure, or exposure measured as continuous variable)?**
   (1) No, outcomes were aggregated (e.g., increase and decrease in flow were combined in an outcome like "changes in flow volume")
   (2) Yes, into categories (e.g., "increase" vs. "decrease")
   (3) Yes, into details (e.g., "needed 20+ pads," qualitative interviews)

7. **Were the outcome measures (dependent variables) clearly defined, *valid, reliable*, and implemented consistently across all study participants?**
   (1) No OR yes, but they allowed for bias (e.g., recall bias, self-report bias)
   (2) No OR yes, but some measures were taken to correct them (e.g., asking for *documented* side effects/disturbance)
   (3) Yes

8. **Were key potential confounding variables measured and adjusted statistically for their impact on the relationship between exposure(s) and outcome(s)?**
   (1) No
   (2) Yes, at least one (excluding vaccine brand and number of doses)
   (3) Yes, multiple
