# Supplementary material for: Menstrual disturbance associated with COVID-19 vaccines: A comprehensive systematic review and meta-analysis
Source: PLoS One. 2025 May 16;20(5):e0320162. doi: 10.1371/journal.pone.0320162 (PMC12083795; doi:10.1371/journal.pone.0320162)
Supplement: S4 Table — (PDF) [file pone.0320162.s005.pdf]

Supplemental Table 4. Primary Source Data Extraction

All the following studies (n=17) were eligible for inclusion in this systematic review, as confirmed in S2 Table. Excluded articles (n=43) are also identified and explained in S2 Table.

First author, publication year (journal) [# in references]	Risk estimate/ mean change in days (95% confidence interval)	Outcome/ appears in	Name of extractor	Date of data extraction
Alvergne, 2022 (Frontiers in Reproductive Health) [17]	1.21 (1.06-1.39)	Figure 2(A): Risk of change in menstrual cycle length associated with any COVID-19 vaccine [first dose]	Sangyal Dorjee	September 4, 2023
	1.34 (1.12-1.59)	Figure 2(A): Risk of change in menstrual cycle length associated with any COVID-19 vaccine [second dose]		
	0.87 (0.54-1.47)	Figure 2(C): Risk of change in menstrual cycle length associated with Moderna vaccine		
	1.13 (0.73-1.75)	Figure 3(A): Risk of increase in menstrual flow volume associated with the first dose of COVID-19 vaccines		
	0.78 (0.45-1.35)	Figure 3(B): Risk of increase in menstrual flow volume associated with the second dose of COVID-19 vaccines		
Alvergne, 2023 (iScience) [16]	1.26 (0.93-1.71)	Figure 2(E): Risk of change in menstrual cycle length associated with AstraZeneca vaccine [first dose]	R.C. Sadoff	November 24, 2023
	1.36 (0.94-1.98)	Figure 2(E): Risk of change in menstrual cycle length associated with AstraZeneca vaccine [second dose]		
	0.96 (0.85-1.10)	Figure 3(A): Risk of increase in menstrual flow volume associated with the first dose of COVID-19 vaccines		
	0.99 (0.85-1.15)	Figure 3(C): Risk of unexpected or intermenstrual bleeding associated with the first dose of COVID-19 vaccines		
Blix, 2022 (Science Advances) [33]	4.7 (3.8-5.7)	Figure 3(C): Risk of unexpected or intermenstrual bleeding associated with the first dose of COVID-19 vaccines [risk estimate for within 28 days of first dose]	Kunchok Dorjee	September 25, 2023
	0.5 (0.4-0.9)	Figure 3(C): Risk of unexpected or intermenstrual bleeding associated with the first dose of COVID-19 vaccines [risk estimate for 28 days after the first dose and before the second dose]		
	4.2 (3.3-5.2)	Figure 3(D): Risk of unexpected or intermenstrual bleeding associated with the second dose of COVID-19 vaccines [risk estimate for within 28 days of second dose]		
	1.0 (0.7-1.3)	Figure 3(D): Risk of unexpected or intermenstrual bleeding associated with the second dose of COVID-19 vaccines [risk estimate for 28+ days after second dose]		
	3.0 (2.0-4.4)	Figure 3(E): Risk of post-menopausal bleeding associated with the first dose of COVID-19 vaccines [risk estimate for within 28 days of first dose]		
	0.6 (0.3-1.2)	Figure 3(E): Risk of post-menopausal bleeding associated with the first dose of COVID-19 vaccines [risk estimate for 28+ days after the first dose and before the second dose]		
	2.2 (1.4-3.5)	Figure 3(F): Risk of post-menopausal bleeding associated with the second dose of COVID-19 vaccines [risk estimate for within 28 days of second dose]		
	0.8 (0.4-1.4)	Figure 3(F): Risk of post-menopausal bleeding associated with the second dose of COVID-19 vaccines [risk estimate for 28+ days after second dose]		

Bouchard, 2022 (Journal of Women's Health) [22]	–0.50 days (–1.31-0.31)	Figure 2(F): Mean difference in length of the first menstrual cycle after receiving the first dose of COVID-19 vaccines	R.C. Sadoff	October 23, 2023
	–0.50 days (–1.36-0.36)	Figure 2(H): Mean difference in length of the second menstrual cycle after receiving the first or second dose of COVID-19 vaccines		
Caspersen, 2023 (Vaccine) [23]	1.15 (1.05-1.27)	Figure 2(A): Risk of change in menstrual cycle length associated with any COVID-19 vaccine	Sangyal Dorjee	September 4, 2023
		Figure 2(B): Risk of change in menstrual cycle length associated with Pfizer-BioNTech vaccine		
	1.60 (1.43-1.80)	Figure 3(A): Risk of increase in menstrual flow volume associated with the first dose of COVID-19 vaccines		
	1.06 (0.92-1.23)	Figure 3(C): Risk of unexpected or intermenstrual bleeding associated with the first dose of COVID-19 vaccines		
Darney et al., 2023 (BJOG) [34]	1.03 (1.01-1.06)	Figure 3(A): Risk of increase in menstrual flow volume associated with the first dose of COVID-19 vaccines	Kunchok Dorjee	November 30, 2023
	1.05 (1.02-1.08)	Figure 3(B): Risk of increase in menstrual flow volume associated with the second dose of COVID-19 vaccines		
Edelman, 2022 (BMJ Medicine) [4]	0.72 days (0.50-0.94)	Figure 2(F): Mean difference in length of the first menstrual cycle after receiving the first dose of COVID-19 vaccines	R.C. Sadoff	October 23, 2023
	0.55 days (0.30-0.79)	Figure 2(G): Mean difference in length of the first menstrual cycle after receiving the second dose of COVID-19 vaccines		
	–0.11 days (–0.41-0.19)	Figure 2(H): Mean difference in length of the second menstrual cycle after receiving the first or second dose of COVID-19 vaccines		
Edelman, 2022 (Obstetrics & Gynecology) [3]	0.64 days (0.27-1.01)	Figure 2(F): Mean difference in length of the first menstrual cycle after receiving the first dose of COVID-19 vaccines	Sangyal Dorjee	September 4, 2023
	0.79 days (0.40-1.18)	Figure 2(G): Mean difference in length of the first menstrual cycle after receiving the second dose of COVID-19 vaccines		
Gibson, 2022 (NPJ Digital Health) [6]	2.16 (1.16-4.03)	Figure 2(A): Risk of change in menstrual cycle length associated with any COVID-19 vaccine [J&J]	Kunchok Dorjee	September 25, 2023
		Figure 2(D): Risk of change in menstrual cycle length associated with Janssen vaccine		
	1.13 (0.87-1.48)	Figure 2(A): Risk of change in menstrual cycle length associated with any COVID-19 vaccine [first dose of mRNA]		
		Figure 2(B): Risk of change in menstrual cycle length associated with Pfizer-BioNTech vaccine [first dose, includes both Pfizer-BioNTech and Moderna]		
		Figure 2(C): Risk of change in menstrual cycle length associated with Moderna vaccine [first dose, includes both Pfizer-BioNTech and Moderna]		
	1.08 (0.83-1.43)	Figure 2(A): Risk of change in menstrual cycle length associated with any COVID-19 vaccine [second dose of mRNA]		
		Figure 2(B): Risk of change in menstrual cycle length associated with Pfizer-BioNTech vaccine [second dose, includes both Pfizer-BioNTech and Moderna]		
		Figure 2(C): Risk of change in menstrual cycle length associated with Moderna vaccine [second dose, includes both Pfizer-BioNTech and Moderna]		
Hariton, 2023 (Fertility and Sterility) [8]	0.04 days (–0.14-0.22)	Figure 2(F): Mean difference in length of the first menstrual cycle after receiving the first dose of COVID-19 vaccines	Kunchok Dorjee	November 10, 2023
	–0.07 days (–0.33-0.19)	Figure 2(H): Mean difference in length of the second menstrual cycle after receiving the first or second dose of COVID-19 vaccines		

Kajiwara, 2023 (Journal of Infection and Chemotherapy) [9]	–0.30 days (–1.38-0.78)	Figure 2(F): Mean difference in length of the first menstrual cycle after receiving the first dose of COVID-19 vaccines	Kunchok Dorjee	November 10, 2023
	1.60 days (–0.68-1.88)	Figure 2(G): Mean difference in length of the first menstrual cycle after receiving the second dose of COVID-19 vaccines		
Ljung, 2023 (BMJ) [15]	1.14 (0.86-1.50)	Figure 3(C): Risk of unexpected or intermenstrual bleeding associated with the first dose of COVID-19 vaccines [risk estimate for 1-7 days after first dose]	Farima R. Mansour	November 29, 2023
	1.01 (0.88-1.16)	Figure 3(C): Risk of unexpected or intermenstrual bleeding associated with the first dose of COVID-19 vaccines [risk estimate for 8-90 days after first dose]		
	0.73 (0.47-1.13)	Figure 3(D): Risk of unexpected or intermenstrual bleeding associated with the second dose of COVID-19 vaccines [risk estimate for 1-7 days after second dose]		
	1.04 (0.89-1.22)	Figure 3(D): Risk of unexpected or intermenstrual bleeding associated with the second dose of COVID-19 vaccines [risk estimate for 8-90 days after second dose]		
	1.15 (0.98-1.35)	Figure 3(E): Risk of post-menopausal bleeding associated with the first dose of COVID-19 vaccines [risk estimate for 1-7 days after first dose]		
	1.14 (1.06-1.25)	Figure 3(E): Risk of post-menopausal bleeding associated with the first dose of COVID-19 vaccines [risk estimate for 8-90 days after first dose]		
	0.98 (0.81-1.19)	Figure 3(F): Risk of post-menopausal bleeding associated with the second dose of COVID-19 vaccines [risk estimate for 1-7 days after second dose]		
	1.14 (1.03-1.25)	Figure 3(F): Risk of post-menopausal bleeding associated with the second dose of COVID-19 vaccines [risk estimate for 8-90 days after second dose]		
Loggia et al., 2023 (Minerva Obstetrics & Gynecology) [32]	4.10 days (3.09-5.10)	Figure 2(H): Mean difference in length of the second menstrual cycle after receiving the first or second dose of COVID-19 vaccines	Farima R. Mansour	November 29, 2023
Suh-Burgmann, 2022 (American Journal of Obstetrics & Gynecology) [31]	1.08 (1.05-1.12)	Figure 3(E): Risk of post-menopausal bleeding associated with the first dose of COVID-19 vaccines [risk estimate within 16 weeks of any dose of vaccine]	R.C. Sadoff	November 24, 2023
		Figure 3(F): Risk of post-menopausal bleeding associated with the second dose of COVID-19 vaccine [risk estimate within 16 weeks of any dose of vaccine]		
	1.05 (1.02-1.08)	Figure 3(E): Risk of post-menopausal bleeding associated with the first dose of COVID-19 vaccines [risk estimate between 17-34 weeks after any dose of vaccine]		
		Figure 3(F): Risk of post-menopausal bleeding associated with the second dose of COVID-19 vaccine [risk estimate within 16 weeks of any dose of vaccine]		
Trogstad, 2023 (Vaccine) [21]	1.07 (0.97-1.17)	Figure 2(A): Risk of change in menstrual cycle length associated with any COVID-19 vaccine [first dose]	Kunchok Dorjee	November 30, 2023
	1.24 (1.13-1.37)	Figure 2(A): Risk of change in menstrual cycle length associated with any COVID-19 vaccine [second dose]		
	1.10 (0.98-1.23)	Figure 2(B): Risk of change in menstrual cycle length associated with Pfizer-BioNTech vaccine [first dose]		
	1.22 (1.06-1.42)	Figure 2(B): Risk of change in menstrual cycle length associated with Pfizer-BioNTech vaccine [second dose]		
	1.01 (0.85-1.19)	Figure 2(C): Risk of change in menstrual cycle length associated with Moderna vaccine [first dose]		
	1.26 (1.11-1.44)	Figure 2(C): Risk of change in menstrual cycle length associated with Moderna vaccine [second dose]		
	1.08 (0.57-2.03)	Figure 2(E): Risk of change in menstrual cycle length associated with AstraZeneca vaccine		
	1.90 (1.69-2.13)	Figure 3(A): Risk of increase in menstrual flow volume associated with the first dose of COVID-19 vaccines		

	1.84 (1.66-2.03)	Figure 3(B): Risk of increase in menstrual flow volume associated with the second dose of COVID-19 vaccines		
	1.09 (1.01-1.17)	Figure 3(C): Risk of unexpected or intermenstrual bleeding associated with the first dose of COVID-19 vaccines		
	1.49 (1.37-1.62)	Figure 3(D): Risk of unexpected or intermenstrual bleeding associated with the second dose of COVID-19 vaccines		
Wang, 2022 (American Journal of Obstetrics & Gynecology) [7]	1.27 (0.98-1.65)	Figure 2(A): Risk of change in menstrual cycle length associated with any COVID-19 vaccine	R.C. Sadoff	October 23, 2023
	1.28 (0.99-1.67)	Figure 2(B): Risk of change in menstrual cycle length associated with Pfizer-BioNTech vaccine		
	1.24 (0.94-1.63)	Figure 2(C): Risk of change in menstrual cycle length associated with Moderna vaccine		
	1.43 (0.85-2.39)	Figure 2(D): Risk of change in menstrual cycle length associated with Janssen vaccine		
Wesselink, 2023 (Vaccine) [24]	1.70 (1.28-2.25)	Figure 2(A): Risk of change in menstrual cycle length associated with any COVID-19 vaccine [first dose]	Kunchok Dorjee	November 30, 2023
	1.22 (0.82-1.82)	Figure 2(A): Risk of change in menstrual cycle length associated with any COVID-19 vaccine [second dose]		
	1.26 days (0.08-2.44)	Figure 2(F): Mean difference in length of the first menstrual cycle after receiving the first dose of COVID-19 vaccines		
	0.95 (0.69-1.30)	Figure 3(A): Risk of increase in menstrual flow volume associated with the first dose of COVID-19 vaccines [at first follow-up]		
	0.74 (0.54-0.99)	Figure 3(A): Risk of increase in menstrual flow volume associated with the first dose of COVID-19 vaccines [at second follow-up]		
